# Jian Yun Qing Hua Decoction inhibits malignant behaviors of gastric carcinoma cells via COL12A1 mediated ferroptosis signal pathway

**DOI:** 10.1186/s13020-023-00799-5

**Published:** 2023-09-12

**Authors:** Baoxinzi Liu, Yu Li, Yuanyuan Xu, Weiwei Xue, Zhichao Jin

**Affiliations:** Department of Medical Oncology, Jiangsu Province Hospital of Chinese Medicine, Affiliated Hospital of Nanjing University of Chinese Medicine, Nanjing, 210029 China

**Keywords:** Jian Yun Qing Hua Decoction, Traditional Chinese medicine, Gastric cancer, Ferroptosis, Stemness

## Abstract

**Background:**

Jian Yun Qing Hua Decoction (JYQHD), a traditional Chinese medicine decoction, which has been applied in the treatment of gastric cancer (GC). We attempt to confirm the anti-gastric cancer effect of JYQHD and explore the mechanism of JYQHD.

**Methods:**

Acute toxicity test was used to understand the toxicity of JYQHD. We studied the expression and prognostic outcome of COL12A1 within GC tissues through the network databases. Using several web-based databases, we analyzed the major components and targets of JYQHD, as well as known therapeutic targets in gastric cancer. The Venn diagram was utilized to obtain the overlapped genes. Lentiviral vector, shRNAs and plasmids, were used to transfect GC cells. Cell counting kit-8 (CCK8), sphere formation, malondialdehyde (MDA), glutathione (GSH), reactive oxygen species (ROS), Fe^2+^, transmission electron microscopy (TEM), quantitative Real-Time Polymerase Chain Reaction (qRT-PCR), Western-Blot (WB), and immunohistochemical (IHC) assays were employed to investigate the role and mechanism of COL12A1 and JYQHD in GC.

**Results:**

The results showed that JYQHD was non-toxic and safe. JYQHD inhibited growth and sphere formation ability through inducing the ferroptosis of GC cells, and suppressed the GC cells induced subcutaneous xenograft tumor growth. COL12A1 was highly expressed in gastric cancer tissues, indicating poor prognosis. COL12A1 specifically enhanced GC cell progression and stemness via suppressing ferroptosis. JYQHD down-regulated COL12A1 in order to suppress the stemness of GC cells via inducing ferroptosis.

**Conclusion:**

COL12A1 inhibited ferroptosis and enhanced stemness in GC cells. JYQHD inhibited the development of GC cells by inhibiting cancer cell stemness via the ferroptosis pathway mediated by COL12A1.

**Supplementary Information:**

The online version contains supplementary material available at 10.1186/s13020-023-00799-5.

## Background

Gastric cancer, a commonly seen cancer, usually displays poor survival and dismal prognosis [1]. Despite the great advances in treatment approaches, GC treatment remains a complex clinical challenge due to tumor recurrence and drug resistance [2, 3]. Cancer stem cells (CSCs) are related to tumor recurrence and chemotherapy-resistant among GC cases, resulting in a high mortality rate [4]. In the context of cancer theory, the CSCs properties confer epithelial-mesenchymal transition (EMT) and treatment resistance to tumor cells [5, 6]. In addition, cancer cells derived from EMT can obtain the stemness and display remarkable treatment resistance [7]. Thus, targeting CSCs has led to the novel anti-tumor therapeutic strategies [8, 9].

Relative to the non-tumor cells, tumor cells display higher demands for iron. This iron dependence makes iron-dependent cells more susceptible to necrosis, a phenomenon known as ferroptosis [10, 11]. Ferroptosis can be induced through ROS accumulation because of iron overload and antioxidant defense system failure, eventually inducing lipid peroxide accumulation. Cystine is a raw material to synthesize GSH. Through the glutamate/cystine antiporter system xc-, glutamate can be transferred into extracellular space and cystine can be transferred into intracellular space. This system is constituted by the single-pass transmembrane anchoring protein solute carrier family 3 member 2 (SLC3A2) and the twelve-pass transmembrane catalytic subunit solute carrier family 7 member 11 (SLC7A11) [12, 13]. GSH is an essential cofactor of glutathione peroxidase 4 (GXP4), which is used to transform lipid peroxides into normal lipids and prevent lipid peroxide accumulation. When glutathione production is insufficient and GPX4 is then inhibited, the ability to remove lipid peroxides is impaired [14]. This incident can induce accumulation of lipid peroxides, leading to oxidative stress damage, and ferroptosis [15]. In general, abnormal lipid metabolism and ROS generation make critical effects on modulating ferroptosis [16, 17]. Therefore, ferroptosis is a potential therapeutic target [18]. Recently, ferroptosis has been suggested to mediate CSCs characteristics among diverse cancer types [19, 20]. Activating ferroptosis for fighting against cancer is a treatment with high safety and selectivity, which has attracted wide attention [21]. However, its underlying molecular mechanism remains to be further explored.

The pathogenesis of GC is very complicated, which involves several genes and signaling pathways. The abnormal gene expression is indicated to be associated with tumor progression and poor prognostic outcome [22]. Therefore, identifying the highly-specific genes for diagnosing GC and predicting its prognosis is of great importance. Collagen type XII α1 chain (COL12A1), which can be encoded via the gene located on 6q12-q13, belongs to fibril-associated collagen family possessing the abnormal triple-helical collagen domains [23]. COL12A1 has attracted great attention because of its promising activities in cancers, since the over-expressed level of COL12A1 can be detected in multiple cancer. As revealed by differentially expressed genes (DEGs) obtained through microarray analysis, COL12A1 shows high expression level within kidney cancer [24]. In addition, COL12A1 expression significantly elevates within ovarian cancer cells with doxorubicin and cisplatin resistance compared with parental cells, which predicts dismal overall survival (OS) [25]. COL12A1 is recognized to be the desmoplastic biomarker in the differentiation of myofibroblasts within GC, indicating the relation between COL12A1 overexpression and tumor malignancy [26]. Moreover, according to GC prognosis analysis, COL12A1 overexpression is related to dismal OS. Based on the above findings, COL12A1 is important for GC, which can be adopted for predicting the prognosis among GC patients.

Traditional Chinese Medicine (TCM) is frequently used for treating cancer in the East [27, 28]. In-vivo and in-vitro experiments have confirmed that TCM extracts exhibit anticancer activities. Astragalus membranaceus extract has been demonstrated to hinder breast cancer cell growth while inducing their apoptosis via the PI3K/AKT/mTOR pathway [29]. Astragalus polysaccharide, a major component in astragalus membranaceus, can sensitize cervical cancer cell line HeLa to cisplatin while improving the anticancer activity of apatinib in the growth, apoptosis and migration of pancreatic cancer cells [30]. Atractylenolides, the essential components of rhizoma atractylodes macrocephala, can induce human colorectal cancer cell apoptosis by activating the mitochondria-dependent pathway [31]. Besides, it can inhibit tumorigenesis of breast cancer through suppressing the nuclear factor-κB (NF-κB) pathway mediated by Toll-Like Receptor 4 (TLR4) [32]. Recent studies have verified that diosgenin extracted from the rhizome of dioscorea plants exhibits diverse pharmacological effects, including anti-tumor activity [33]. According to in-vivo and in-vitro experiments performed by Yifan Qian et al., coix seed extract has synergistic activity in enhancing the effect of gemcitabine on treating pancreatic cancer [34]. Tanshinones, an isolated extract from salvia miltiorrhiza, is confirmed with an anti-tumor impact on liver cancer and colon cancer [35, 36]. As suggested in another study, zedoariae rhizoma possibly makes a critical effect on inhibiting melanoma through activating macrophage function [37]. Radix bupleuri-derived saikosaponin D is suggested to hinder the proliferation of triple-negative breast cancer cells through β-catenin pathway [38]. In conclusion, Chinese herbal extracts and active ingredients exhibit anti-tumor effects, and it is necessary to develop the naturally derived anticancer drugs [39]. Jian Yun Qing Hua Decoction (JYQHD), a TCM prescription, is composed of *Pseudostellaria heterophylla* (Miq.) Pax (Tai zi shen), *Astragalus membranaceus* (Fisch.) Bunge (Huang qi), *Atractylodes macrocephala* Koidz. (Bai zhu), *Dioscorea polystachya* Turcz. (Shan yao), *Coix lacryma-jobi* L. (Yi yi ren), *Salvia miltiorrhiza* Bge. (Dan shen), *Curcuma phaeocaulis* Valeton (E zhu), *Citrus aurantium* L. (Zhi shi), *Bupleurum chinense* DC. (Chai hu), and so on. JYQHD is extensively used in the clinical treatment of esophageal, gastric and colorectal cancer, and it is suggested to lower the risk of recurrence, retard tumor growth, prevent tumor metastasis, enhance the patient quality of life and relieve complications. Zhenhua Guan et al. identified that Tanshinone IIA from salvia miltiorrhiza could suppress the proliferation of GC via inducing p53 upregulation-mediated ferroptosis [40]. Haiwei Ni et al. revealed that Tanshinone IIA could inhibit the stemness of gastric cancer cells through inducing ferroptosis [41]. A few studies have reported that Chinese herbal extracts can induce ferroptosis in gastric cancer cells. Therefore, this study aimed to examine the impact of JYQHD on modulating GC cell growth, ferroptosis and stemness. Furthermore, this study investigated whether JYQHD exerts its anti-tumor effect through regulating the ferroptosis pathway by targeting COL12A1 in GC cells.

## Methods

### Preparation of JYQHD decoctions

Herbal formula of JYQHD comprised: 15 g of *Pseudostellaria heterophylla* (Miq.) Pax (Tai zi shen), 30 g of *Astragalus membranaceus* (Fisch.) Bunge (Huang qi), 30 g of *Atractylodes macrocephala* Koidz. (Bai zhu), 30 g of *Dioscorea polystachya* Turcz. (Shan yao), 45 g of *Coix lacryma-jobi* L. (Yi yi ren), 15 g of *Salvia miltiorrhiza* Bge. (Dan shen), 10 g of *Curcuma phaeocaulis* Valeton (E zhu), 10 g of *Citrus aurantium* L. (Zhi shi), 10 g of *Bupleurum chinense* DC. (Chai hu), 30 g of *Hedyotis diffusa* Willd. (Bai hua she she cao), 30 g of *Scutellaria barbata* D.Don (Ban zhi lian), 30 g of *Lobelia chinensis* Lour. (Ban bian lian), and 15 g of *Paris polyphylla* Sm. (Chong lou). The boiling of crude drugs was performed with a boiling device. At first, the drugs were soaked in 1000 mL double-distilled water for 1 h and then boiled to 100 ℃ for 2 h, after which the residue was boiled with 1000 mL double-distilled for 2 h water again. Next, the two extracts were mixed and concentrated to 3 g/mL and then filtered through a 0.2 μm filter. The extracts were stored at − 20 ℃ until use.

### Preparation of JYQHD-medicated serum and Identification of major chemical components

We acquired Sprague Dawley (SD) rats weighing 235 ± 35 g from Hunan STA Laboratory Animal Technology Co. Ltd., (license number SCXK (Xiang) 2019–0004). These SD rats were raised in the clean environment under the following conditions, 20–24 ℃ temperature, 50–60% humidity and the 12-h/12-h light/dark cycle, with free access to food and water. The JYQHD quality was controlled in line with the Standard Operation Procedure of Chinese Pharmacopoeia. In the meanwhile, JYQHD samples were obtained from the Chinese Pharmacy of the Affiliated Hospital of Nanjing University of Chinese Medicine, Jiangsu Province Hospital of Chinese Medicine. JYQHD herbs underwent a series of processing, including soaking, boiling, and concentration to 3 g/mL. Thereafter, SD rats were randomized into JYQHD and the control groups, with 10 each. The animals were given intragastric administration of 54 g/kg JYQHD at 1 ml/100 g body weight twice a day for 5 days consecutively, which doubled the equivalent conversion dose for the 70 kg adult. Animals in the control group were given gavage of distilled water. At 3 h after the final gavage, each rat was given intraperitoneal injection of 1% pentobarbital sodium for anesthesia. Bloods were sampled from abdominal aorta, followed by centrifugation at 3000 rpm for a 15 min to collect serum. Afterwards, the current experiment inactivated the serum by water bath at 56 ℃ for 30 min, and kept at − 20℃ for later use. Subsequently, the JYQHD-medicated serum was diluted with RPMI-1640 medium to the expected doses [42].

**Fig. 1:**
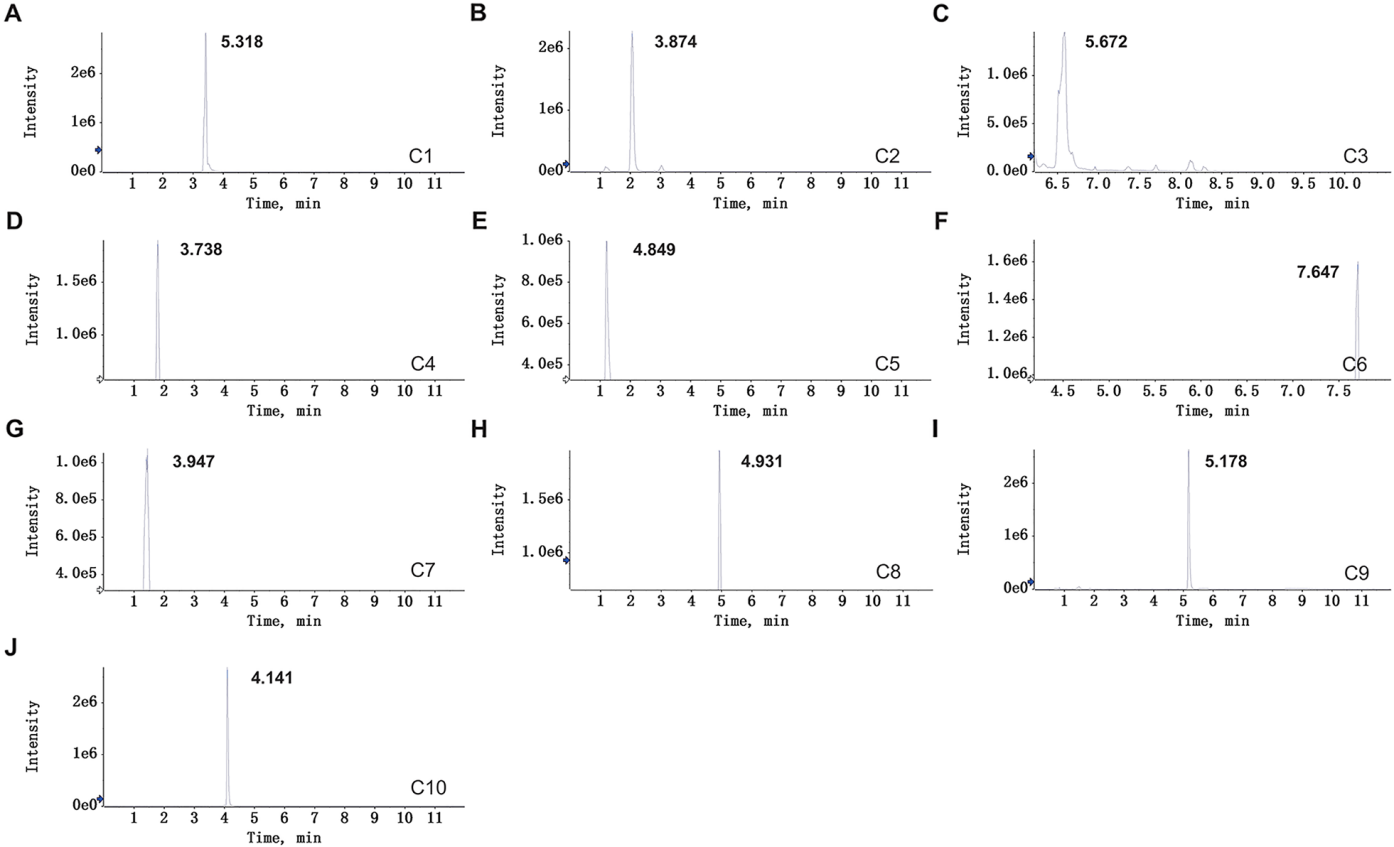
Typical extracted ion chromatograms of the major constituents in JYQHD-medicated serum. JYQHD-medicated serum using liquid chromatography/time-of-flight mass spectrometry. C1: Myricitrin ([M + H]^+^, *m/z* 464.095); C2: D-fructose ([M−H]^−^, *m/z* 179.056); C3: Naringin ([M−H]^−^, *m/z* 579.168); C4: Nobiletin ([M + H]^+^, *m/z* 403.138); C5: Scutellarin ([M−H]^−^, *m/z* 461.071); C6: Adenosine ([M + H]^+^, *m/z* 268.104); C7: Tangeritin ([M + H]^+^, *m/z* 373.127); C8: Ononin ([M + H]^+^, *m/z* 431.134); C9: Hesperetin ([M + H]^+^, *m/z* 611.195); C10: Luteolin ([M−H]^−^, *m/z* 461.071)

The major components in JYQHD-medicated serum were identified based on the liquid chromatography/time-of-flight mass spectrometry (LC-TOFMS) technology. In addition, the series 1290 HPLC system (Agilent, USA) was utilized for quantification in combination with Triple TOFTM 5600 mass spectrometer (AB SCIEX, Foster City, CA). The Agilent Poroshell 120 SB-C18 column was utilized for separation under 35 ℃ at the 0.3 ml/min flow rate. The mobile phase included ultrapure water and acetonitrile/H2O (v/v, 95/5, B) including 0.125% formic acid (v/v) and 10 mM ammonium formate (v/v). To be specific, the mobile phase included 15–21% B within 0–2 min; 21% B within 2–4 min; 21–23% B within 4–5 min; 23% B within 5–8 min; 23–24% B within 8–13 min; 24–40% B within 13–16 min; 40–80% B within 16–17 min; 80–100% B within 17–19 min; 100% B within 19–23 min; 100–15% B within 23–26 min; 15% B within 26–30 min. Each sample was kept under 4 ℃. MS detection was carried out to use Triple TOFTM 5600 (AB SCIEX, Foster City, CA) under positive/negative ion modes (ion spray voltage floating, 5500/4500 V, separately), using electron spray ionization source. TOFMS scan parameters were shown below: accumulation time, 0.25 s; TOF mass range, m/z 100–1000; heater temperature, 550 ℃; ion source gases 1/2, 60 psi; curtain gas, 35 psi; collision energy, 10 eV; declustering potential, 80 V; with high-sensitivity and DBS options being set. Characteristic XIC chromatograms for 10 main components of JYQHD-medicated serum were displayed in Fig. 1 and Table 1. Finally, the 10 main contents of Myricitrin, D-fructose, Naringin, Nobiletin, Scutellarin, Adenosine, Tangeritin, Ononin, Hesperetin and Luteolin in the JYQHD-medicated serum were determined.

**Table 1:** The structure of the standards in LC-TOFMS chromatograms of JYQHD-medicated serum

Name	Molecular formula	Molecular weight	Structures
Myricitrin	C21H20O12	464.38	
D-fructose	C6H12O6	180.16	
Naringin	C27H32O14	580.53	
Nobiletin	C21H22O8	402.39	
Scutellarin	C21H18O12	462.36	
Adenosine	C10H13N5O4	267.24	
Tangeritin	C20H20O7	372.37	
Ononin	C22H22O9	430.40	
Hesperetin	C28H34O15	610.56	
Luteolin	C15H10O6	286.24	

### Cells and culture

We acquired human gastric epithelial cells (GES-1) in Beina Chuanglian Biotechnology Research Institute (Beijing, China), while human GC cells (BGC-823, SGC-7901 and MKN-45) in the Type Culture Collection, Chinese Academy of Sciences (Shanghai, China). The above cell lines were kept in the RPMI-1640 medium (Gibco, Waltham, MA, USA) that contained 1% penicillin–streptomycin (PS) and 10% fetal calf serum (FCS) (Gibco) as well as incubated in the humid incubator under 37 ℃ and 5% CO_2_ conditions.

### Cell treatment

GC cells (MKN-45 and SGC-7901) were exposed to JYQHD-medicated serum at specific doses. Moreover, MKN-45 and SGC-7901 cells were subject to treatment with ferroptosis antagonist Ferrostatin-1 (Fer-1) (Sigma-Aldrich, St. Louis, MO, USA) before and during the treatment with JYQHD-medicated serum.

### The Gene Expression Profiling Interactive Analysis (GEPIA) database

GEPIA has been developed as the web-based platform for exploring collagen family genes mRNA expression levels and overall survival in cancer and healthy samples from TCGA and Genotype Tissue Expression (GTEx) databases with gastric cancer patients.

### The Cancer Genome Atlas (TCGA) and the Gene Expression Omnibus (GEO) datasets

Stomach adenocarcinoma (STAD) gene expression patterns were acquired from TCGA network database [43] and GEO network database [44]. In TCGA, the RNA-sequencing (RNA-seq) count data related to STAD, and matched clinical data were obtained using the gdc-client.exe [45]. A total of 407 STAD samples were obtained, including 375 tumor samples and 32 normal samples. The data were merged by Perl language, and Ensembl ID names were converted to the gene symbol matrix based on the Ensembl database [46]. It was indicated by edgeR package tutorial that, a gene having a low read count was generally not the interested gene used in subsequent analysis. Therefore, in this study, genes that had count per million (cpm) ≧ 1 were retained. Genes were filtered by the edgeR package rpkm function [47]. The receiver operating characteristic (ROC) curve is a comprehensive index which can be used to reflect the sensitivity and specificity of continuous variables. Finally, the gene expression, survival probability and the ROC curve of COL12A1 were extracted. To confirm the expression of COL12A1 creditability, GSE13911 and GSE66229 datasets in GEO were used for validation. Those STAD normalized gene expression patterns from GSE13911 and GSE66229 in GEO were also obtained by the R package limma, and GPL570 platform was used to analyze these data Affymetrix Human Gene 1.0 ST Array. Afterwards, each probe was transformed into gene symbol using the perl soft. Meanwhile, the median level of related probes for a certain gene was calculated to remove those replicated probes. The expression of COL12A1 in each dataset was extracted to verify the previous results.

### Human Protein Atlas (HPA) and Kaplan–Meier (KM) plotter databases

In this study, the COL12A1 protein level within GC and healthy gastric tissues were examined by HPA. Then, the association of COL12A1 with survival of GC cases is frequently evaluated by a network tool Kaplan–Meier plotter, which is the web-based platform for analyzing RNA-sequencing and mRNA Affymetrix Genechip datasets among GC cases. The data was collected based on online databases.

### Screening the main active compounds of JYQHD

The active compounds of JYQHD were originated from the Traditional Chinese Medicine Systems Pharmacology (TCMSP) database [48]. To better screen the active compounds of JYQHD, OB 30% [49] and DL 0.05 [50] were applied to be screening situations in this study.

### Collection of targets

Based on the PubChem database [51], the Swiss Target Prediction website was used to expect all the potential targets of the active compounds in JYQHD by setting Homo sapiens. In addition, the relevant targets in GC were acquired using the DisGeNET, GeneCards, and Online Mendelian Inheritance in Man (OMIM) databases. The Venn diagram was obtained via the Venn diagram web tool [52], aiming to visualize the overlapping targets.

### Gene Ontology (GO) as well as Kyoto Encyclopedia of Genes and Genomes (KEGG) analysis on overlapping targets

R software clusterProfiler function was adopted for conducting GO and KEGG analysis to examine biological functions of genes within the overlapping targets, upon the *P* < 0.01 and FDR < 0.05 thresholds.

### Lentiviral vector, shRNA, plasmids, and cell transfection

The specific shRNAs against COL12A1 (sh-COL12A1, GAT CGG CAA TAC TCT CAC AGG CAT GGC TCG AGC CAT GCC TGT GAG AGT ATT GCT TTT TTG GAA TTC) and corresponding control shRNA (sh-NC, UUC UCC GAA CGU GUC ACG UTT) as well as pcDNA 3.1/COL12A1 and its empty vector were obtained from Genechem Co., Ltd. (Shanghai, China). Subsequently, the constructed plasmids were co-transfected with package plasmids into 293 T cells by Lipofectamine 3000 (Invitrogen, CA, USA). After 48 h, the lentivirus particles were collected in the supernatant and MKN-45 or SGC-7901 cells were infected with lentivirus for 96 h. The efficiency of infection was determined by qRT-PCR and WB assays.

### Acute toxicity assay

The BALB/c mice were randomized as 6 groups, with 6 males and 6 females in each group. Before oral administration, the animals experienced a 12-h fasting period, with free access to water. After the detection, the TCM extract was filtered with the mesh screen and concentrated till the maximal level reached 5 g/ml (a limit set in order not to hinder oral administration). In addition, animals in each group were given oral administration of JYQHD at 1, 2, 3, 4 and 5 (maximal dose) g / ml, respectively. By contrast, the control animals were given an equivalent amount of water. From the initial 1 to 14 days after the first administration, each animal was monitored and the mouse mortality rate was recorded. LD50 was used as the acute toxicity index, and its value together with corresponding 95% confidence limits (CL) was measured through the Bliss’s approach.

### Tumor xenograft assay

Our animal experimental protocols were approved by Animal Ethics and Research Committee of Nanjing University of Chinese Medicine. In brief, we obtained the 5-week-old BALB/c female nude mice in Beijing Weitong Lihua Experimental Animal Technical Co., Ltd. (Beijing, China). To analyze the effect of JYQHD or COL12A1 on tumorigenicity of GC cells, each BALB/c nude mouse was given subcutaneous inoculation with transfected or untransfected MKN-45 or SGC-7901 cells. 7 days after subcutaneous injection in nude mice, mice were randomized as several groups (n = 6 in each group). Thereafter, mice were given oral administration of physiological saline or 39 g/kg JYQHD once daily for 19 consecutive days. Finally, after animal sacrifice, tumor tissues were collected and weighed; tumor volume was calculated by volume = (length × width^2^)/2 [53].

### CCK8 assay

CCK-8 assay (Sigma-Aldrich, St. Louis, MO, USA) was used for the detection of cell proliferation. In brief, cells were digested using 0.25% trypsin (NCM Biotech, Suzhou, China). Thereafter, cells (5 × 10^3^/well) were cultivated within each well of the 96-well plate. Then, overnight incubation was performed under 37° C. After treated with JYQHD-medicated serum or 5-fluorouracil (5-FU), all wells were introduced with CCK-8 solution (10 µL) to incubate under 37° C for 2 h. Afterwards, cells were lysed with DMSO (150 μL), while the absorbance (OD) value at 490 nm was measured using the ELx800 microplate reader (BioTek, Winooski, VT, USA). Finally, we determined cell growth inhibition rate below: Inhibition rate = (1−OD_experiment_/OD_control_) × 100%.

### Sphere-formation assay

Briefly, the transfected or untransfected MKN-45 or SGC-7901 cells (500/well) were cultured into the 6-well plates with ultralow attachment with DMEM/F12 medium consisting of 20 ng/mL EGF, 20 ng/mL FGF and 2% B27, but without stem-cell-specific serum. Then, cells were incubated with JYQHD-medicated serum, Fer-1 at the required concentration under 37 °C and 5% CO_2_ conditions for 10 days. The phase-contrast microscope was employed to observe sphere formation.

### Fe^2+^ level measurement

The iron assay kit (Sigma-Aldrich, St. Louis, MO, USA) was used to determine ferrous iron (Fe^2+^) level. To be specific, the transfected or untransfected MKN-45 or SGC-7901 cells (2 × 10^6^/well) were inoculated into the 6-well plates, followed by exposure to JYQHD-medicated serum, Fer-1 for 24 h. Then, cells were harvested and rinsed, followed by measurement of Fe^2+^ content in line with the specific protocols. Finally, the multifunctional enzyme label analyzer (PE Enspire, USA) was employed to measure absorbance value at 593 nm.

### MDA and GSH measurements

The transfected or untransfected MKN-45 or SGC-7901 cells (2 × 10^6^/well) were inoculated into the 6-well plates, followed by exposure to JYQHD-medicated serum, Fer-1 for 24 h. Thereafter, the commercially available MDA Detection Kit (Sigma-Aldrich, St. Louis, MO, USA) was used to measure intracellular MDA level. In brief, after corresponding treatment, cells were subject to lysis for collecting supernatants, which were then exposed to 15 min treatment using MDA reaction solution (200 μl) under 100 ℃. Thereafter, the microplate reader was utilized to determine MDA content at 532 nm. The commercially available GSH Detection Kit (Sigma-Aldrich, St. Louis, MO, USA) was used for detecting intracellular GSH content at the OD value of 420 nm. Each procedure was performed following specific protocols.

### ROS determination

The transfected or untransfected MKN-45 or SGC-7901 cells (2 × 10^6^/well) were inoculated into the 6-well plates, followed by 24 h exposure to JYQHD-medicated serum, Fer-1. 2ʹ,7ʹ-dichlorodihydrofluorescein diacetate (DCFH-DA; 20 µM, Sigma-Aldrich, St. Louis, MO, USA), the cell-penetrating probe, was added to determine intracellular ROS level. Following 30 min incubation, the spectrofluorimeter was used to measure fluorescence intensity at the emission and excitation wavelengths of 530 and 488 nm, respectively. Then, cells were directly placed under a fluorescence microscope and photographed in line with the specific protocols.

### TEM assay

After 24 h, the optical microscope was employed for observing distance and mitochondrial morphology of the pre-treated MKN-45 or SGC-7901 cells. In brief, 3% glutaraldehyde was added to fix cells, followed by post-fixation with 1% osmium tetroxide, gradient ethanol dehydration, as well as Duruban resin embedding. Thereafter, this study mounted thin sections onto the copper grids, followed by uranyl acetate/lead citrate staining and observation with SEM (HITACHI-600, Japan) to observe the alterations of cell and mitochondrial morphology.

### qRT-PCR assay

The pre-treated MKN-45 or SGC-7901 cells were digested within trypsin, followed by homogenization. Thereafter, cellular RNA was extracted with TRIzol reagent (Invitrogen, Carlsbad, CA, USA). Subsequently, total RNA was prepared into cDNA using the TaKaRa RT reagent (Invitrogen, Carlsbad, CA, USA) kit via reverse transcription. The following thermocycling conditions were set, 5 min under 95 °C; 10-s under 95 °C, 10-s under 95 °C as well as 30-s under 60 °C for totally 40 cycles. Afterwards, the ABI 7500 fast qRT-PCR system was utilized to measure gene expression levels using the DNA-binding dye SYBR Green and the 2^−ΔΔCq^ method [54], with GAPDH being an internal reference. The following primer pairs were used for real-time PCR: COL1A1, forward, 5′-GAG GGC CAA GAC GAA GAC ATC-3′ and reverse, 5′-CAG ATC ACG TCA TCG CAC AAC-3′; COL4A1, forward, 5′-TGT TGA CGG CTT ACC TGG AGA C-3′ and reverse, 5′-GGT AGA CCA ACT CCA GGC TCT C-3′; COL12A1, forward, 5′-CCA CAG GTT CAA GAG GTC CC-3′ and reverse, 5′-TGT GTT AGC CGG AAC CTG GA-3′; Oct4 forward 5ʹ-GGA TTG GCT TCG TCA TCA CT-3ʹ, reverse 5ʹ-ATA ATC AAC CCG CGG TAC TC-3ʹ; Sox2 forward 5ʹ-GGA TTG GCT TCG TCA TCA CT-3ʹ, reverse 5ʹ-ATA ATC AAC CCG CGG TAC TC-3ʹ; GPX4 forward 5ʹ-AGA GAT CAA AGA GTT CGC CG-3ʹ, reverse 5ʹ-TTG TCG ATG AGG AAC TGT GG-3ʹ; SLC7A11 forward 5ʹ-GGA TTG GCT TCG TCA TCA CT-3ʹ, reverse 5ʹ-ATA ATC AAC CCG CGG TAC TC-3ʹ; GAPDH forward 5ʹ-TGT TCG TCA TGG GTG TGA AC-3ʹ; reverse 5ʹ-ATG GCA TGG ACT GTG GTC AT-3ʹ.

### WB assay

After being washed by pre-chilled PBS, RIPA buffer was supplemented into the pre-treated MKN-45 or SGC-7901 cells for 15 min on ice. Thereafter, the lysate was centrifuged at 12,000 ×*g* and 4 °C for 15 min. The Bradford method was used to determine protein level, and the samples were preserved under − 80 °C before the subsequent analysis. Thereafter, proteins (20 μg) were split through 10–12% SDS-PAGE, followed by transfer on the PVDF membranes (Bio-Rad, Hercules, CA, USA). The PVDF membrane was subject to blocking using the dried skimmed milk and incubation with primary antibodies. After blocking with bovine serum albumin (BSA) for 1 h, membranes were incubated overnight with primary antibodies under 4 °C. Subsequently, additional 2 h incubation was performed using HRP-labeled secondary antibodies under room temperature. The chemiluminescence analyzer (Amersham Biosciences, Boston, MA, USA) was utilized for blot visualization. During the experiments, we used the following antibodies: anti-COL12A1 antibody (1:2000, ab121304 Abcam), anti-Oct4 antibody (1:1000, ab181557, Abcam), anti-Sox2 antibody (1:1500, ab171380, Abcam), anti-SLC7A11 antibody (1:1500, ab175186, Abcam), anti-GPX4 (1:2000, ab125066, Abcam), and anti-β-actin antibody (1:1000, ab8227, Abcam). The ECL detection kit (Yeasen, Shanghai, China) was used for determining protein signals, with β-actin being an internal reference.

### IHC assay

After formaldehyde fixation and paraffin embedding for 48 h at room temperature, the paraffin-embedded tissues were sliced into cross-sections for deparaffinization. Then, cells were incubated using 3% H_2_O_2_ under room temperature for 10 min to block the activity of endogenous peroxidase. After being rinsed by PBS for 5 min three times, tissue sections were further incubated using primary antibodies under 4 °C overnight, followed by PBS rinsing three times, and later, 2 h incubation using HRP-labeled secondary antibodies under room temperature. Afterwards, the DAB chromogenic reagent was used to visualize the antibody-binding sites in dark. Thereafter, the tissue sections were rinsed with distilled water for 5 min three times, followed by 5 min hematoxylin counter-staining, 5 min differentiation using 1% hydrochloric acid ethanol, 2 min dehydration with 95% ethanol twice, and 5 min transparentizing with xylene. Then, the nuclei were sealed with neutral resin and examined under an inverted microscope. Three regions were chosen randomly and the microscope was used to take photographs. Positive cells were denoted as pale yellow, tan, and dark brown.

### Statistical analysis

One-way ANOVA was used for statistical analysis using SPSS24.0. Results were represented to be mean ± SD. *P* < 0.05 and *P* < 0.01 represented statistical significance and extreme significance, respectively.

## Results

### Acute toxicity of JYQHD

In the acute toxicity test of mice, no death case was reported, even in the maximum dosage group. Therefore, the median lethal dose (LD50) was not measured. At the same time, there was no clinical manifestation related to drug administration, indicating that the drug was not toxic and its clinical dosage was safe. The results of acute toxicity test were displayed in Additional file 4: Table S1.

### JYQHD reduced the in-vitro viability of MKN-45 and SGC-7901 cells

**Fig. 2:**
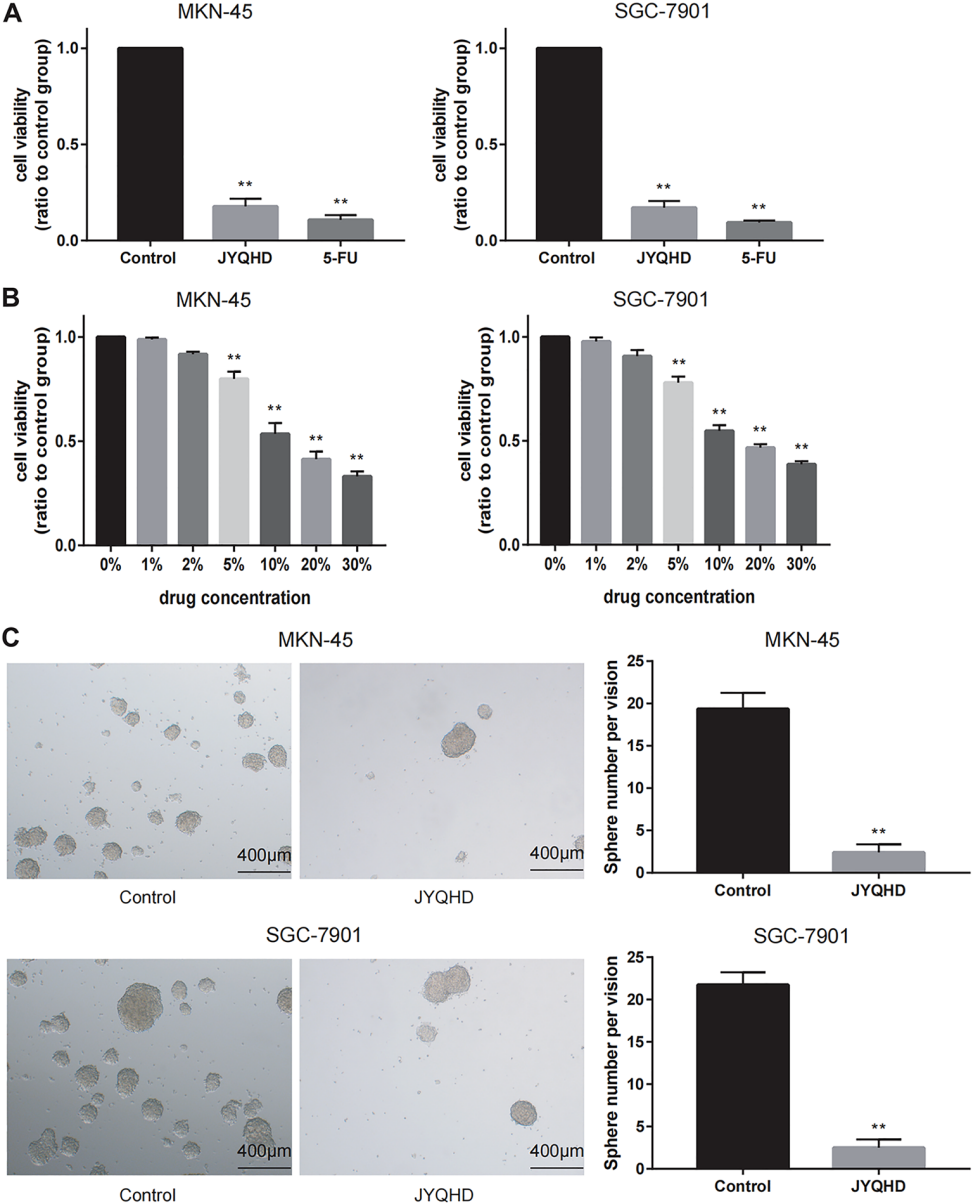
JYQHD inhibits the growth of GC cell lines. **A** Both JYQHD-medicated serum (100%) and 5-FU were used to treat MKN-45 and SGC-7901 cells for 48 h, and the viability of GC cells was inhibited in both groups. **B** JYQHD-medicated serum at diverse contents was used to treat MKN-45 and SGC-7901 cells for a 24 h period, and inhibition rate was determined through CCK8 assay. A 10% concentration of JYQHD-medicated serum was defined as an IC50 value. **C** JYQHD-medicated serum suppressed stemness of GC cells. 10% concentration of JYQHD-medicated serum was added to treat MKN-45 and SGC-7901 cells, and the size and number of sphere was measured by sphere-formation assay. **P < 0.01 JYQHD group or 5-FU group vs the control group. Scale bar = 400 μm (**C**)

CCK8 assays were performed to assess the viability of GC cells after exposure to JYQHD-medicated serum (100%) and 5-Fu (5 µg/mL) for 48 h. The experimental results showed that JYQHD could attenuate the viability of gastric cancer cells and 5-FU (Fig. 2A). Therefore, in the follow-up experiment, we chose JYQHD as the research object. To find the IC50 value of JYQHD, CCK8 assay was performed to assess the inhibition ratio of GC cells after exposure to JYQHD-medicated serum at different doses (30%, 20%, 10%, 5%, 2%, 1% and 0%) for 24 h. After JYQHD-medicated serum treatment, MKN-45 and SGC-7901 cell lines showed the dose-dependent reductions in cell viability relative to the control cells. As revealed by CCK8 analysis, JYQHD suppressed MKN-45 and SGC-7901 cell growth, and the IC50 value was approximately 10% concentration of JYQHD-mediated serum (Fig. 2B). Therefore, 10% was chosen as the representative concentration of JYQHD-medicated serum for the following experiments.

### JYQHD impaired the maintenance of MKN-45 and SGC-7901 cell stemness

Accumulating evidence has suggested that cancer cell stemness is responsible for reconstitution and propagation of GC [55]. Sphere forming assay was further adopted for evaluating MKN-45 and SGC-7901 cell stemness. As a result, JYQHD mitigated the sphere forming ability, as evidenced by the reduced sphere count and size (Fig. 2C). JYQHD-medicated serum treatment caused the decreased GC cell stemness relative to the control group.

### JYQHD facilitated the ferroptosis of MKN-45 and SGC-7901 cells

**Fig. 3:**
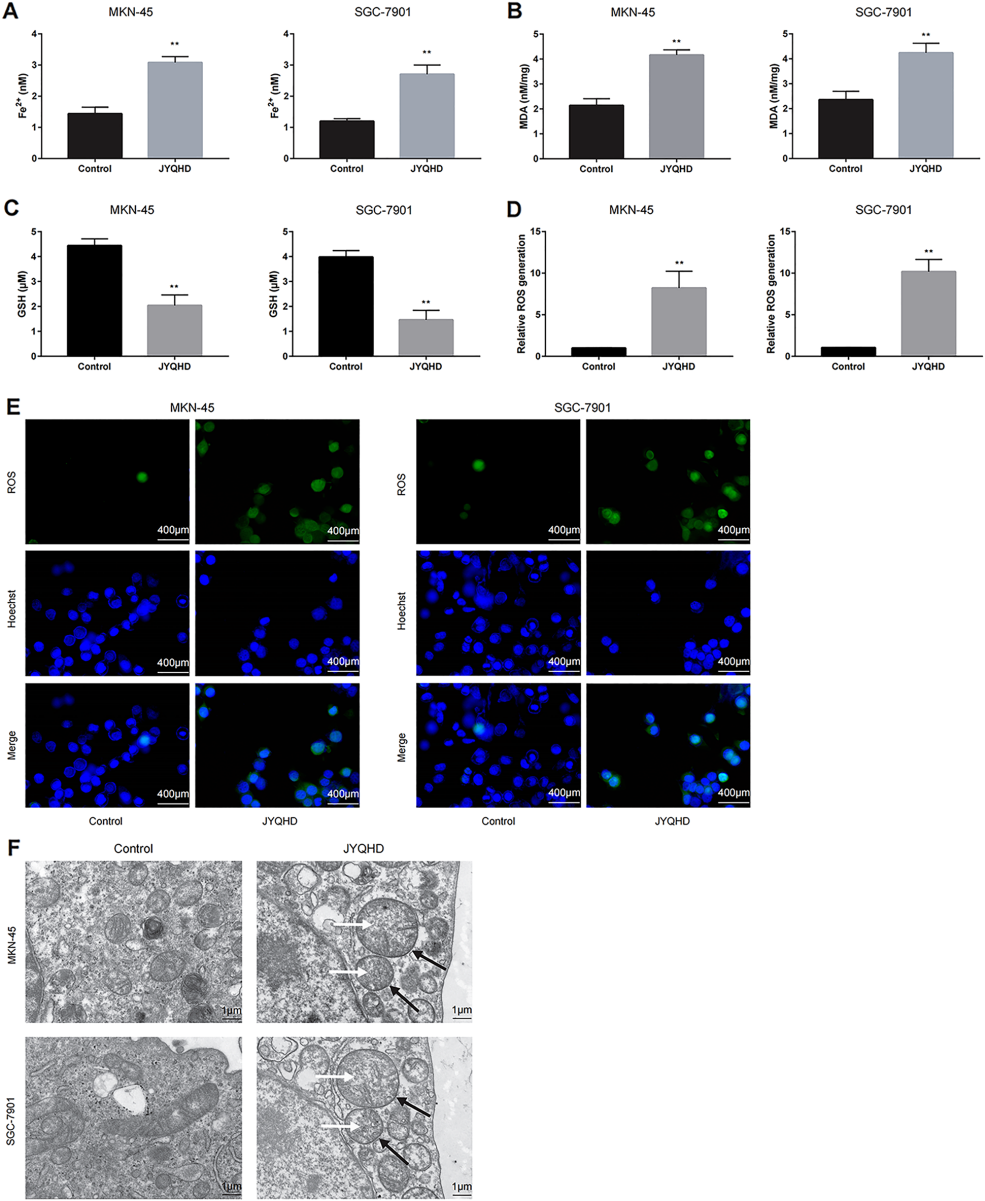
JYQHD promotes GC cell ferroptosis. **A**–**E** JYQHD-medicated serum induced the ferroptosis of GC cells. Ferroptosis was evaluated by detecting Fe^2+^, MDA, GSH and ROS levels. This work acquired confocal laser scanning microscope images for ROS production. (**F**) JYQHD-medicated serum promoted the mitochondrial membrane density increased (black arrow) and the mitochondrial ridge shrank or disappeared (white arrow) in the ferroptosis process of MKN-45 and SGC-7901 cells. This work acquired TEM images showing mitochondrial morphology. **P < 0.01 JYQHD group vs the control group. Scale bar = 400 μm (**E**). Scale bar = 1 μm (**F**)

Ferroptosis represents the special non-apoptotic, iron-dependent programmed cell death mode, with the features of lipid peroxidation-mediated ROS accumulation and inefficient GPX4 [56, 57]. To determine the JYQHD-induced cell ferroptosis mechanism, JYQHD-medicated serum was added to treat MKN-45 and SGC-7901 cells for 24 h. Next, the impacts of JYQHD on the levels of four key ferroptosis features including GSH, ROS, MDA and Fe^2+^ in GC cells were analyzed. Therefore, JYQHD significantly decreased GSH content, but elevated MDA and Fe^2+^ contents within MKN-45 and SGC-7901 cells (Fig. 3A–C). To better analyze the effect of JYQHD on ferroptosis, we further analyzed JYQHD-mediated ROS accumulation with the fluorescence microscope and the multifunctional microplate reader. According to our observations, JYQHD significantly upregulated ROS accumulation within the above two cell lines (Fig. 3D, E). To explore how JYQHD affected mitochondrial morphology, TEM was performed to measure mitochondrial morphology in JYQHD-treated cells (Fig. 3F). Thus, JYQHD significantly mediated mitochondrial crista disappearance within the MKN-45 and SGC-7901 cell lines. Collectively, JYQHD mediated in-vitro ferroptosis of GC cells.

### JYQHD attenuatd GC cell stemness through inducing the ferroptosis signaling pathway

**Fig. 4:**
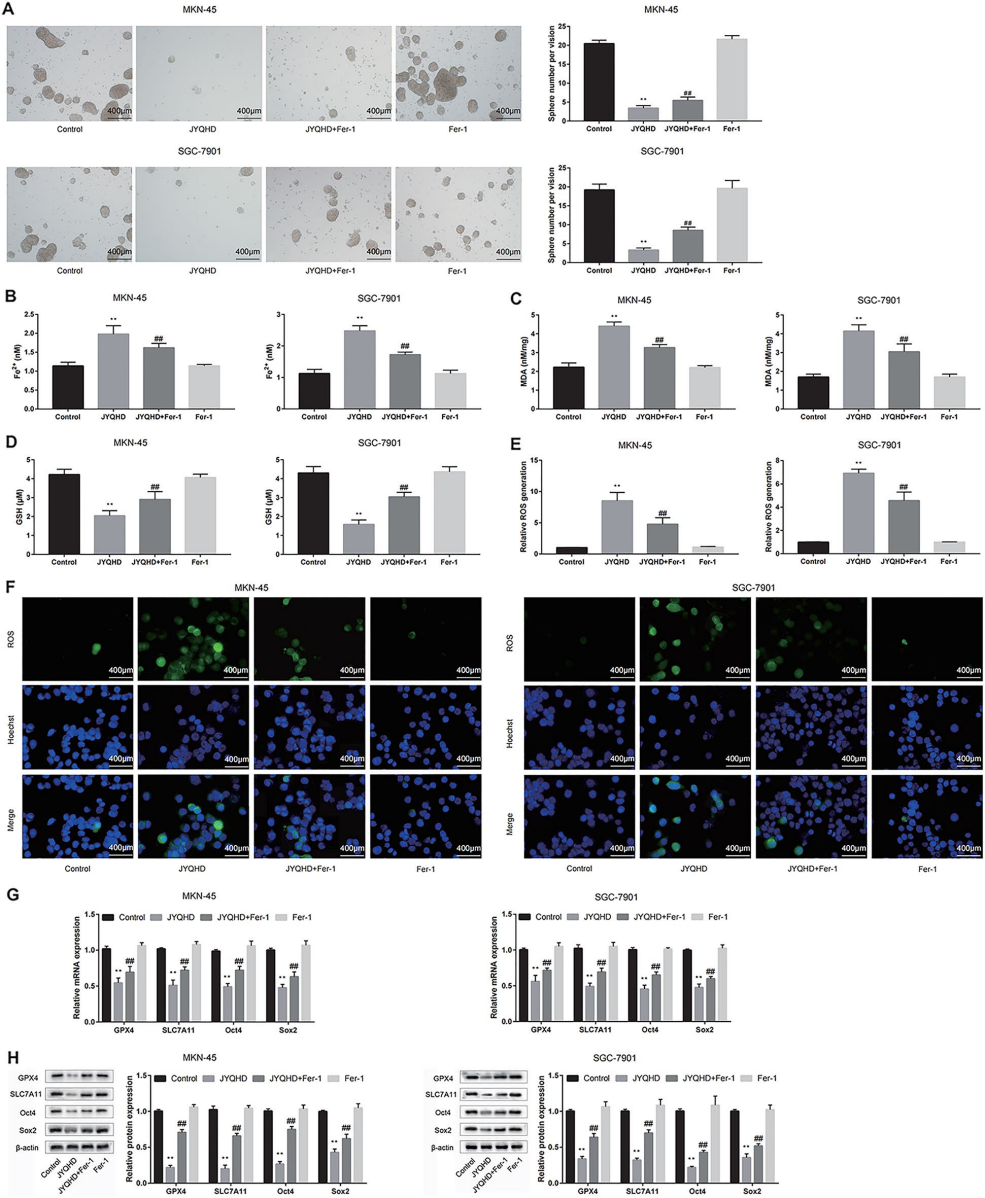
JYQHD inhibits the stemness of GC cells through the ferroptosis pathway. **A**–**F** JYQHD-medicated serum with/without ferroptosis signaling inhibitor Fer-1, and Fer-1 alone were added to treat MKN-45 and SGC-7901 cells. Cancer cell stemness was evaluated by sphere formation assay. Ferroptosis was evaluated by detecting Fe^2+^, MDA, GSH and ROS levels. **G**, **H** The qRT-PCR and WB assays were conducted to detect the pretreated MKN-45 and SGC-7901 cells for assessing stemness and ferroptosis-related markers. **P < 0.01 JYQHD group vs the control group. ^##^*P* < 0.01 JYQHD + Fer-1 group vs JYQHD group. Scale bar = 400 μm (**A**). Scale bar = 400 μm (**F**)

Recently, ferroptosis has been suggested to mediate the stemness [58, 59]. This study further analyzed the JYQHD mechanism of action in GC cell stemness. To better determine the function of ferroptosis pathway underlying the effect of JYQHD on inhibiting cancer cell stemness in GC, the pathway inhibitor Fer-1 was used to examine the presence/absence of the rescue effect. In the course of the experiments, we found that Fer-1 could protect gastric cancer cells and prevent the ferroptosis, but had no promoting effect on gastric cancer cells. As a result, JYQHD attenuated the cancer cell stemness and induced the ferroptosis compared with the control group, while Fer-1 reversed the cancer cell stemness and ferroptosis phenotypes induced by JYQHD (Fig. 4A–F). Based on our qRT-PCR and WB results, GPX4 and SLC7A11 expression was significantly down-regulated in JYQHD-treated group relative to the control group. Similarly, Oct4 and Sox2 expression was down-regulated in JYQHD-treated group. By contrast, Fer-1 rescued the alterations in markers of ferroptosis (GPX4 and SLC7A11) and cancer cell stemness (Oct4 and Sox2) induced by JYQHD (Fig. 4G, H). Collectively, it was suggested that JYQHD inhibited cancer cell stemness by inducing the ferroptosis pathway within GC cells.

### Mining of overlapped targets and enrichment analysis

**Fig. 5:**
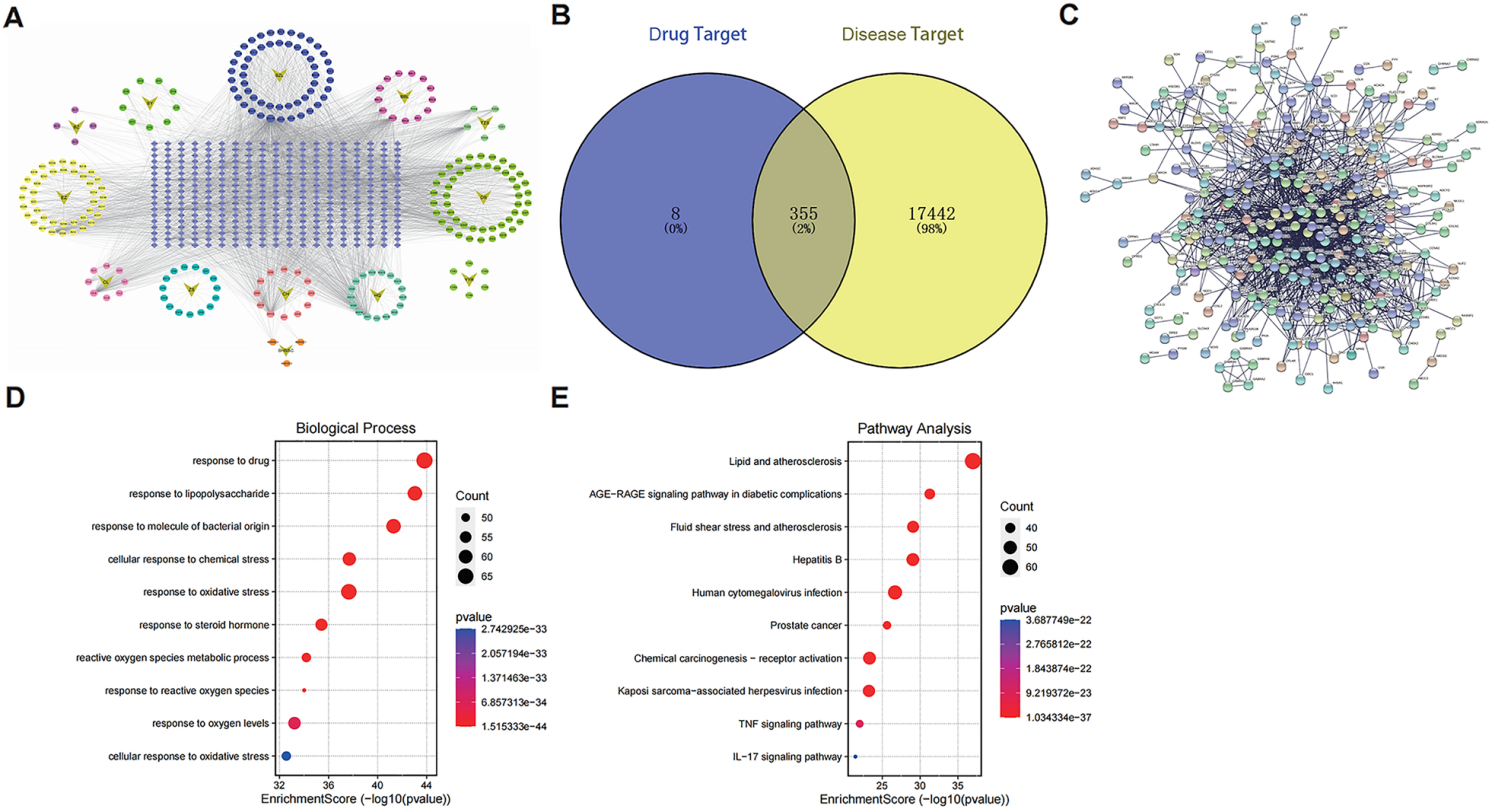
The overlapped genes of bioinformatics analysis between JYQHD and STAD target genes. **A** JYQHD compound-target network. The circle represents the JYQHD active compound, the V-shape figure represents the herb, and the gray diamond represents the target. **B** The Venn diagram of the overlapped targets between the potential targets in JYQHD and the known therapeutic targets for STAD. **C** Protein–protein interaction network of the 355 overlapped genes. **D** GO (BPs) enrichment analysis on 355 overlapped anti-STAD targets. **E** KEGG pathway enrichment analysis on 355 overlapped anti-STAD targets

To acquire the anti-STAD targets in JYQHD, a comparative analysis was performed for the potential targets in JYQHD and the therapeutic targets for STAD. Through the screening of the network pharmacological databases, totally 237 active compounds were obtained, namely, 5 Tai zi shen, 17 Huang qi, 4 Bai zhu, 9 Shan yao, 6 Yi yi ren, 49 Dan shen, 42 E zhu, 15 Zhi shi, 12 Chai hu, 3 Bai hua she she cao, 54 Ban zhi lian, 13 Ban bian lian and 8 Chong lou (Fig. 5A). Finally, a total of 363 targets of active compounds and 17,797 targets of GC were obtained. Based on the results, 355 overlapped genes existed between the potential targets in JYQHD and the known therapeutic targets for STAD (Fig. 5B) (see Additional file 1), which were the anti-STAD targets with high confidence. The protein–protein interaction network of the 355 overlapped genes was presented (Fig. 5C). To deeply investigate the biological mechanisms underlying these 355 therapeutic targets in JYQHD, the GO/KEGG pathway enrichment analysis was performed. The top 10 most significant GO/KEGG pathways associated with these 355 targets were presented (Fig. 5D, E). According to all the results of GO enrichment analysis, we found that these targets were engaged in the following biological processes (BPs), including regulation of reactive oxygen species metabolic process, regulation of response to reactive oxygen species, regulation of lipid metabolic process, membrane lipid metabolic process, iron ion transmembrane transport, cellular iron ion homeostasis and glutathione metabolic process, which were associated with ferroptosis (see Additional file 2). In addition, all the results of KEGG enrichment analysis in the 355 targets showed that, the anti-STAD effect of JYQHD presented the correlation with cancer-associated pathways, followed by Lipid and atherosclerosis, Chemical carcinogenesis-reactive oxygen species and Glutathione metabolism, which were correlated with ferroptosis (see Additional file 3).

### COL12A1 expression and prognostic outcome within GC cells and tissues

**Fig. 6:**
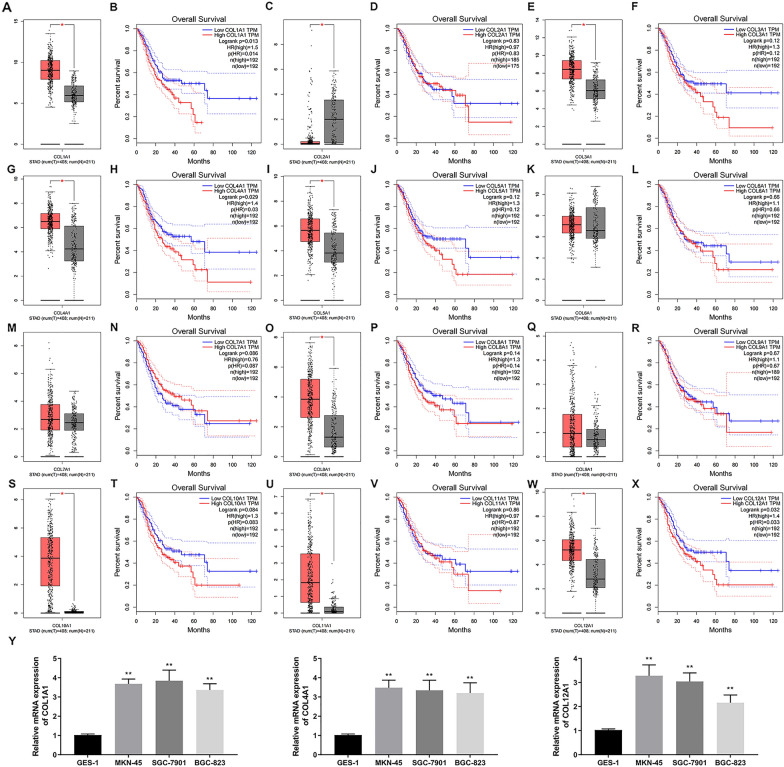
Expression and OS curves of 12 collagen family genes are plotted for GC in GEPIA. Expression on boxplots: **A** COL1A1, **C** COL2A1, **E** COL3A1, **G** COL4A1, **I** COL5A1, **K** COL6A1, **M** COL7A1, **O** COL8A1, **Q** COL9A1, **S** COL10A1, **U** COL11A1, **W** COL12A1. Overall survival on curve diagrams: **B** COL1A1, **D** COL2A1, **F** COL3A1, **H** COL4A1, **J** COL5A1, **L** COL6A1, **N** COL7A1, **P** COL8A1, **R** COL9A1, **T** COL10A1, **V** COL11A1, **X** COL12A1. **Y** The COL1A1, COL4A1 and COL12A1 mRNA expression within GC cells and healthy cells. *P < 0.05, **P < 0.01 GC group vs normal group

**Fig. 7:**
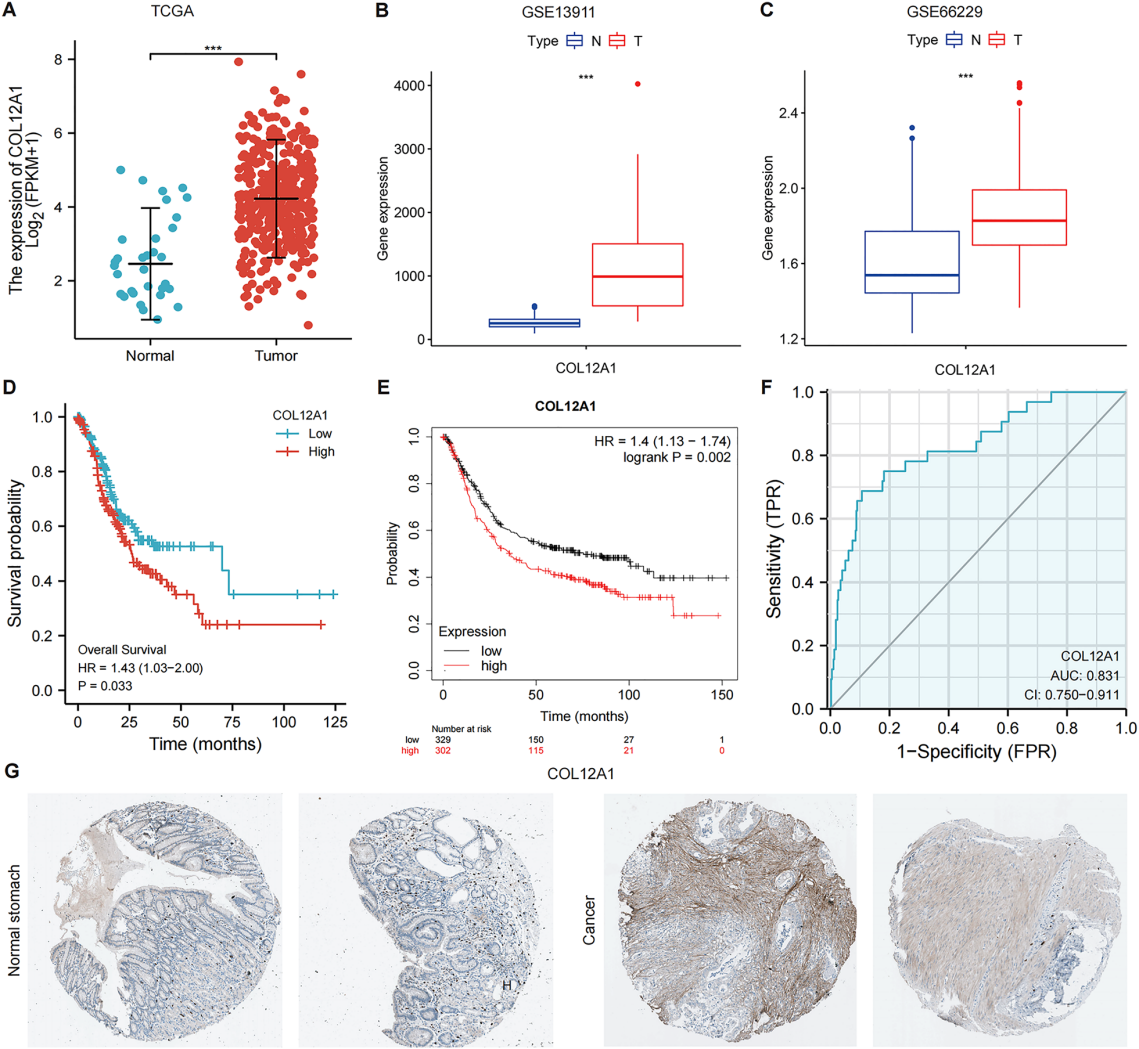
COL12A1 level within GC cells and tissues and its prognostic outcome, diagnostic value. **A**–**C** The expression of COL12A1 within normal and tumor tissues in the stomach in TCGA and GEO datasets. **D**, **E** The overall survival analysis of COL12A1 in gastric cancer patients from the TCGA and KM Plotter datasets. **F** The ROC curve of the COL12A1 gene from the TCGA datasets. **G** The COL12A1 protein level within GC and healthy gastric tissues. This work collected images in HPA datasets. *P < 0.05, **P < 0.01, ***P < 0.001 GC group vs the control group

Among the searched genes, we found that these genes were not differentially expressed in gastric cancer, or there had been considerable related studies in the literature. Therefore, considering that the bioinformatic analysis was based on the existing literatures, we decided to start with family genes. In addition, we found the presence of collagen family genes in 355 overlapping anti-STAD targets, including COL1A1 and COL3A1. Literature search revealed that Collagen family genes might be related to the prognosis of gastric cancer patients [60]. Biao Yang et al. identified that COL1A1, COL1A2, COL5A2, COL6A3 and COL12A1 were potential core genes associated with the progression of stomach adenocarcinoma using bioinformatic analysis [61]. Considering that there were few studies on Collagen family genes in gastric cancer, we performed the bioinformatics analysis of COL1A1, COL2A1, COL3A1, COL4A1, COL5A1, COL6A1, COL7A1, COL8A1, COL9A1, COL10A1, COL11A1 and COL12A1. Through GEPIA database, we found that COL1A1, COL4A1 and COL12A1 was differentially expressed in patients with gastric cancer, significantly suggesting a poor prognosis (Fig. 6A–X). Based on Fig. 6Y, compared with GES-1 cells, COL1A1, COL4A1 and COL12A1 mRNA expression elevated in GC cells (BGC-823, SGC-7901, MKN-45). Based on the number of literatures and the value of the reports, we chose COL12A1 as a gene deserving investigation. To identify COL12A1 expression level in tumor versus non-carcinoma tissue samples, limma was used to analyze TCGA-STAD, GSE13911 and GSE66229 datasets, respectively (Fig. 7A–C). Multiple data revealed that COL12A1 was highly denoted in gastric cancer. In addition, correlations of COL12A1 with overall survival (OS) in STAD were studied through the TCGA and Kaplan–Meier plotter (Fig. 7D, E). Based on the results of the analysis, higher COL12A1 expression predicted the dismal OS of GC cases relative to lower COL12A1 expression, suggesting that COL12A1 made a vital effect on GC development. To determine whether the COL12A1 gene has high diagnostic value for GC patients, this study performed the ROC curve based on the TCGA database to evaluate the diagnostic efficiency of the COL12A1 gene. As shown in Fig. 7F, the area under the curves (AUC) of COL12A1 was 0.831. Subsequently, the result showed that the COL12A1 gene had relatively high diagnostic value for the patients of GC. In addition, HPA-based IHC analysis on GC samples suggested the moderate COL12A1 protein expression within healthy gastric tissues, whereas high expression within GC tissues (Fig. 7G).

### COL12A1 silencing decreased GC cell stemness

**Fig. 8:**
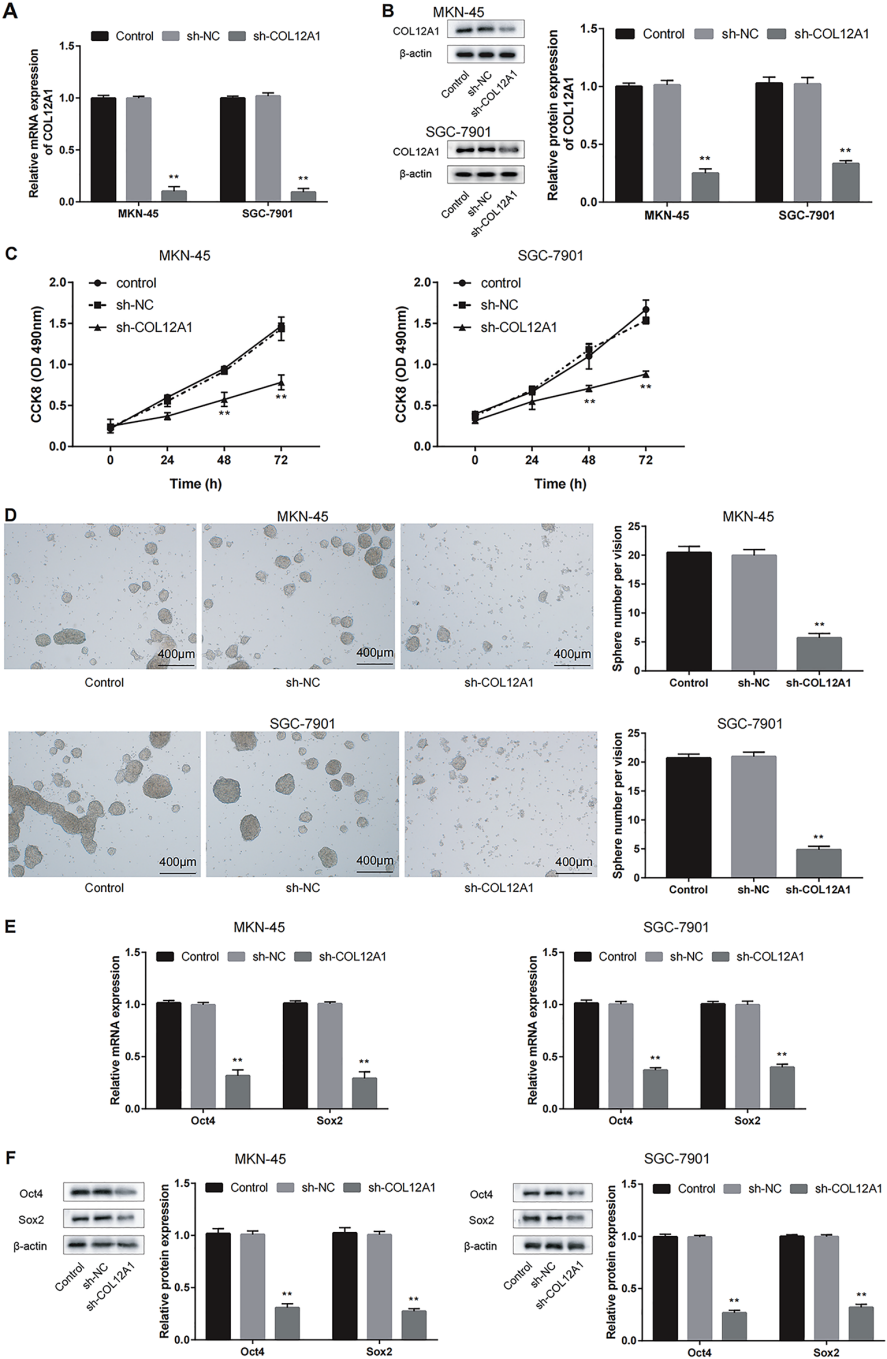
COL12A1 enhances the stemness of GC cells. **A**, **B** MKN-45 and SGC-7901 cells were subject to COL12A1 shRNAs transfection. qRT-PCR and WB assays were conducted to verify the knockdown efficiency of COL12A1. **C** After transfected with COL12A1 shRNAs, GC cell activity was significantly decreased in sh-COL12A1 group. **D** COL12A1 knockdown suppressed the sphere size and number, as evidenced by sphere-formation assay. **E**, **F** The qRT-PCR and WB assays were conducted to detect the transfected MKN-45 and SGC-7901 cells for assessing stemness markers level. **P < 0.01 sh-COL12A1 group vs sh-NC group. Scale bar = 400 μm (**D**)

**Fig. 9:**
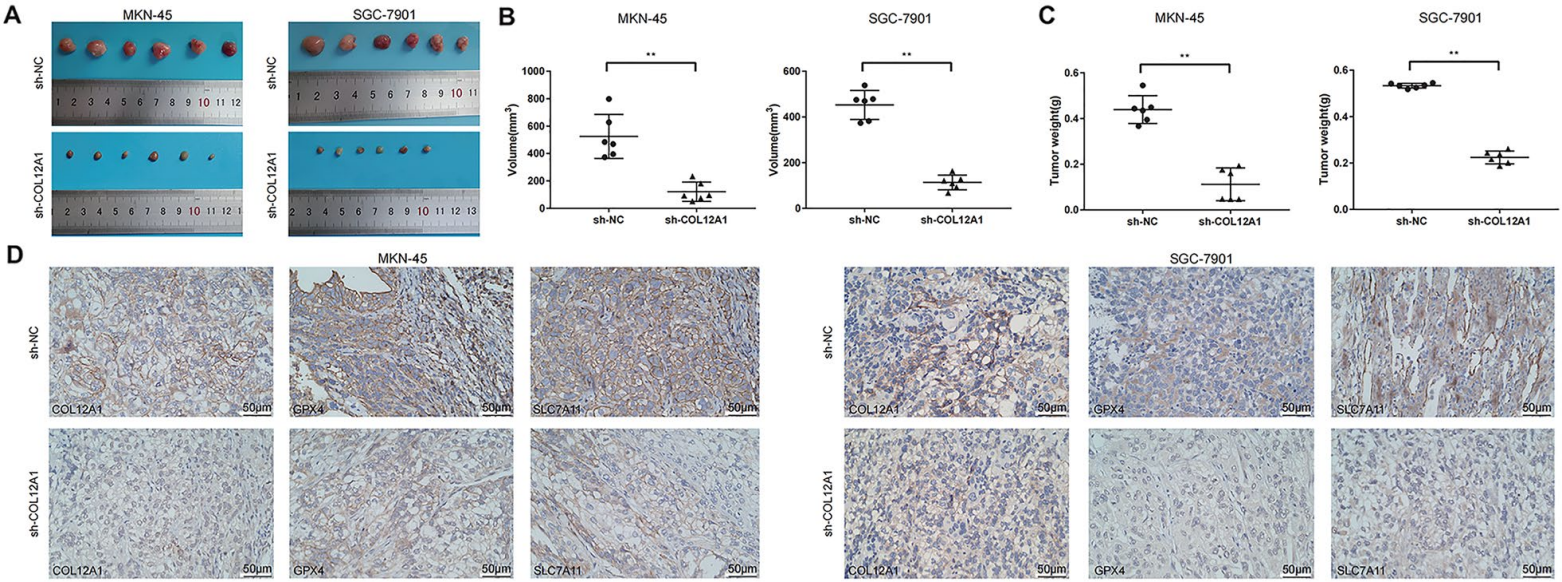
COL12A1 promotes GC cell growth in vivo. **A** Knockdown of COL12A1 suppressed in-vivo xenograft tumor growth. Tumor distinction was shown in the representative photograph through tumor xenograft assay. **B**, **C** The differences in weight and volume of subcutaneous tumors were determined and compared between sh-COL12A1 and sh-NC cells groups. **D** IHC analyzed the expression of COL12A1, GPX4 and SLC7A11 protein of tumors from the sh-NC and sh-COL12A1 groups. **P < 0.01 sh-COL12A1 group vs the control group. Scale bar = 50 μm (**D**)

Therefore, we attempted to explore the role of COL12A1 role in GC cell stemness. The qRT-PCR and WB assays were performed to verify shRNA-mediated COL12A1 knockdown efficiency (Fig. 8A, B). Compared with the control group, COL12A1 knockdown significantly decreased cell activity (Fig. 8C). COL12A1 knockdown mitigated the sphere forming ability, as evidenced by the reduced sphere count and size (Fig. 8D). In addition, COL12A1 knockdown significantly down-regulated the markers for stemness (Oct4 and Sox2) (Fig. 8E, F). We further investigated the role of COL12A1 in the oncogenesis of GC. Animal experiment was performed with a subcutaneous xenograft model. Furthermore, immunohistochemistry was used to detect the protein expression of COL12A1 in the tumors of the two groups. Representative photographs of resceted tumors were shown in Fig. 9A. Consistently, regardless of the identical tumor-forming rate, the COL12A1 knockdown MKN-45 and SGC-7901 cells-derived tumors had reduced tumor volume and weight (Fig. 9B, C). Certainly, the expression of COL12A1 decreased in sh-COL12A1 group (Fig. 9D). In summary, COL12A1 knockdown decreased GC cell stemness.

### Knockdown of COL12A1 induced the ferroptosis process in GC cells

**Fig. 10:**
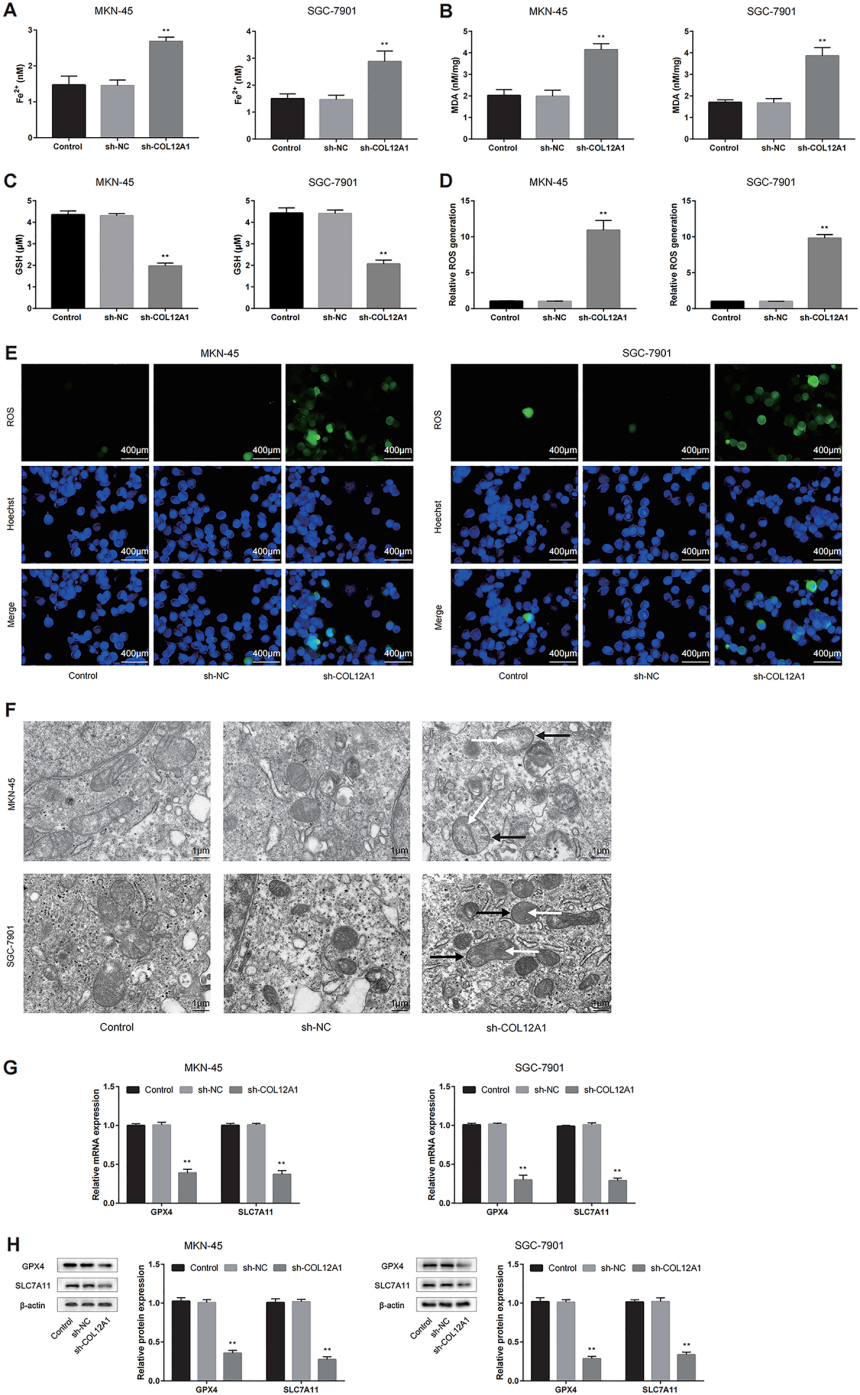
COL12A1 inhibits GC cell ferroptosis. **A**–**E** COL12A1 knockdown promoted GC cell ferroptosis. Ferroptosis was evaluated by detecting ROS, MDA, Fe^2+^ and GSH levels. This work acquired confocal laser scanning microscope images for ROS production. **F** Low-expression of COL12A1 impaired the shape of mitochondrial cristae (white arrow) and promoted the density of mitochondrial membrane (black arrow) in the ferroptosis process of MKN-45 and SGC-7901 cells. This work obtained TEM images for mitochondrial morphology. **G**, **H** The qRT-PCR and WB assays were carried out to detect the transfected MKN-45 and SGC-7901 cells for assessing ferroptosis markers level. **P < 0.01 sh-COL12A1 group vs the control group. Scale bar = 400 μm (**E**). Scale bar = 1 μm (**F**)

Previous studies have indicated that knockdown of COL12A1 can reduce cell viability and stemness of MKN-45 and SGC-7901 cells. Therefore, we further investigated the effects of COL12A1 on the ferroptosis of these two cell lines. The results revealed that COL12A1 knockdown significantly decreased GSH contents, but increased MDA and Fe^2+^ contents within GC cells compared with the control group (Fig. 10A–C). Moreover, decreasing COL12A1 also increased ROS accumulation in MKN-45 and SGC-7901 cells under the fluorescence microscopy in the sh-COL12A1 group (Fig. 10D, E). To establish the impact of COL12A1 on mitochondrial morphology, we subsequently examined the mitochondrial morphology of the GC cells under a TEM. The results showed that the mitochondrial membrane density increased and the mitochondrial ridge shrank or disappeared of GC cell lines in the sh-COL12A1 group (Fig. 10F). In addition, sh-COL12A1 group had significantly reduced GPX4 and SLC7A11 expression relative to the control group in vitro (Fig. 10G, H). Consistently, the expression of GPX4 and SLC7A11 reduced in sh-COL12A1 group in vivo (Fig. 9D). In summary, the above results indicated that COL12A1 inhibited ferroptosis of GC cells.

### JYQHD facilitated the ferroptosis signaling pathway of GC by inhibiting the expression of COL12A1

**Fig. 11:**
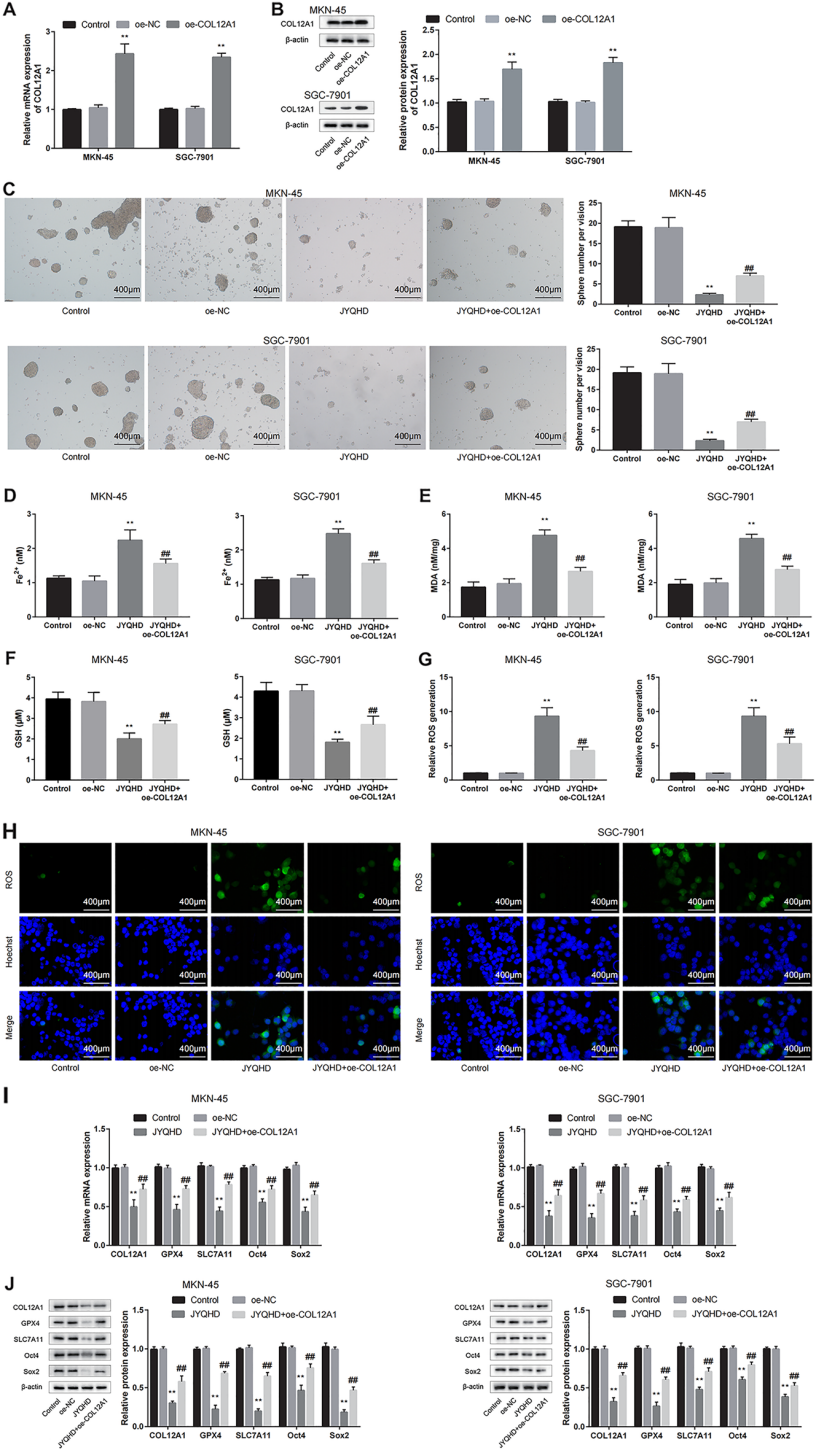
JYQHD inhibits the stemness and induces ferroptosis of GC cells via COL12A1. **A**, **B** COL12A1-plasmids were transfected into MKN-45 and SGC-7901 cells, qRT-PCR and WB assays were conducted to confirm COL12A1 over-expression efficiency. **C** JYQHD-medicated serum was added to treat transfected MKN-45 and SGC-7901 cells, and sphere-formation assay was conducted to analyze the sphere size and number. **D**–**H** Those transfected MKN-45 and SGC-7901 cells were exposed to JYQHD-medicated serum, and ferroptosis was evaluated by detecting Fe^2+^, MDA, GSH and ROS levels. **I**, **J** The qRT-PCR and WB assays were carried out to detect the pretreated MKN-45 and SGC-7901 cells for assessing ferroptosis and stemness related markers expression. **P < 0.01 JYQHD group vs the control group. ^##^*P* < 0.01 JYQHD + oe-COL12A1 group vs JYQHD group. Scale bar = 400 μm (**C**). Scale bar = 400 μm (**H**)

To further illustrate the function of COL12A1 in the mechanism of JYQHD-medicated serum in suppressing GC, the transfected cells were treated with JYQHD-medicated serum. qRT-PCR and WB assays were performed to verify pcDNA3.1 plasmid-mediated COL12A1 overexpression efficiency (Fig. 11A, B). In vitro, based on the previous experimental results, JYQHD-mediated serum promoted the occurrence of ferroptosis and suppressed GC cell stemness (Fig. 11C–H). In addition, JYQHD-medicated serum significantly declined GPX4, SLC7A11 (ferroptosis markers), Sox2, Oct4 (cancer cell stemness markers), and COL12A1 expression, while overexpression of COL12A1 significantly attenuated the effect of JYQHD on promoting the ferroptosis and inhibiting the stemness of GC cells, and enhanced the levels of GPX4 and SLC7A11 (ferroptosis markers), Sox2 and Oct4 (cancer cell stemness markers), and COL12A1 (Fig. 11I, J). In conclusion, the study demonstrated that JYQHD facilitated the ferroptosis signaling pathway to impair stemness by inhibiting the expression of COL12A1 in GC.

### JYQHD suppressed xenograft growth through inhibiting COL12A1 expression and inducing ferroptosis

**Fig. 12:**
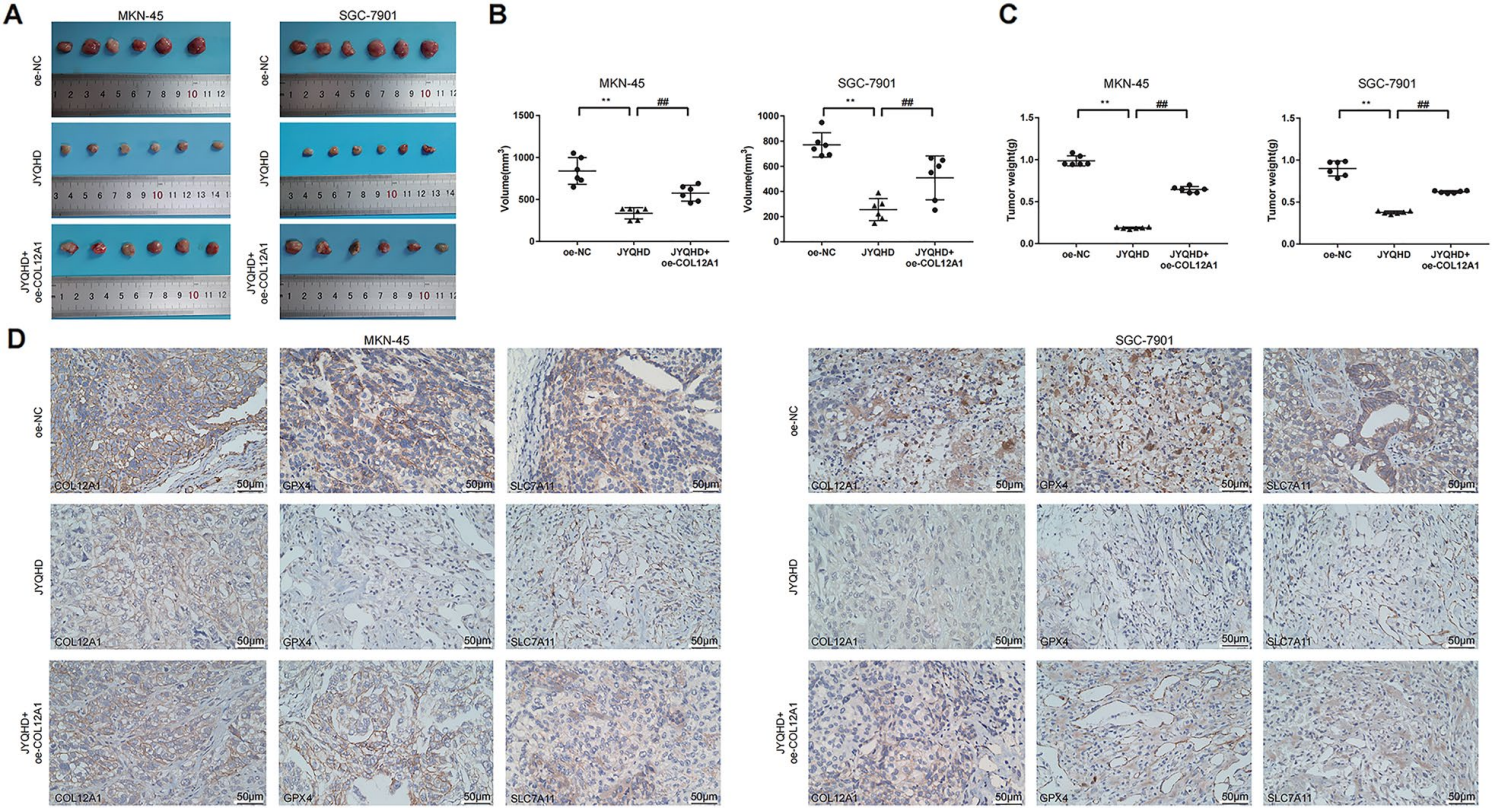
JYQHD inhibits xenograft tumor growth via COL12A1-mediated ferroptosis in GC cells. **A** JYQHD suppressed in-vivo xenograft tumor growth, while COL12A1 over-expression counteracted the anticancer effect of JYQHD. The distinction of tumors was shown in the representative photograph through tumor xenograft assay. **B**, **C** The differences in weight and volume of subcutaneous tumors were determined and compared between oe-NC, JYQHD and JYQHD + oe-COL12A1 groups at 19th day. **D** IHC analyzed the expression of COL12A1, GPX4 and SLC7A11 protein of tumors from the oe-NC, JYQHD and JYQHD + oe-COL12A1 cells groups. **P < 0.01 JYQHD group vs the control group. ^##^*P* < 0.01 JYQHD + oe-COL12A1 group vs JYQHD group. Scale bar = 50 μm (**D**)

Based on a previous assay, tumor xenograft assay indicated that JYQHD effectively inhibited GC growth in vivo, while overexpression of COL12A1 counteracted the anticancer effect of JYQHD, and the tumor mass increased significantly compared with JYQHD group. After animal sacrifices, representative photographs of resected tumors were exhibited (Fig. 12A). Consistently, JYQHD inhibited gastric tumor growth, while oe-COL12A1 attenuated the anti-tumor effect of JYQHD and increased tumor volume and weight compared with JYQHD group (Fig. 12B, C). Immunohistochemical analysis results demonstrated that JYQHD downregulated the expression of COL12A1, GPX4 and SLC7A11 in JYQHD group, while the expression of COL12A1, GPX4 and SLC7A11 increased in JYQHD + oe-COL12A1 group compared to JYQHD group (Fig. 12D). On the whole, JYQHD inhibited the growth of xenograft tumor via COL12A1-mediated ferroptosis in GC cells.

## Discussion

GC is a frequently occurring cancer worldwide, which is characterized by high metastasis and relapse rates. Recent studies have indicated that, activating cancer stemness programs within cancer induces tumor development, metastasis, and treatment resistance, while targeting stemness facilitates to control the refractory disease [62]. Sung-Hwa Sohn et al. demonstrated that foretinib inhibited cancer stemness and gastric cancer cell proliferation by decreasing CD44 and c-MET signaling [63]. Interestingly, ferroptosis, a relatively new discovered mode of cell death, has been found to be a very vital role in GC [64]. Haiwei Ni et al. suggested that the miR-375/SLC7A11 regulatory axis was a potential target to induce ferroptosis and could thus attenuate the stemness of GC cells [65]. Liying Zhao et al. disclosed that apatinib could induce the ferroptosis through GPX4 in the GC cells [66]. Therefore, the potential drugs and genes for inducing ferroptosis need to be studied. TCM was found to prevent the occurrence of GC, control the development and decrease the relapse risk of GC [67–69]. JYQHD, a TCM prescription, which is often used in the treatment of gastric cancer in the clinical practice. In addition, no vitro or vivo studies have confirmed the anti-GC effect of JYQHD, and no studies have focused on explaining the mechanism. Therefore, the anti-GC effect and its mechanism were explored in this study. Currently, it is believed that the components of TCM compound absorbed into blood through oral administration are the effective components that really act on the cells. Meanwhile, the serum of TCM compound provides the stable external environment for the pharmacological application of TCM, which excludes the potential influencing factors in TCM preparation methods. Therefore, the JYQHD-medicated serum was used for in-vitro experiments, and the water extract of JYQHD was adopted for in-vivo experiments [70]. The *in-vitro* results confirmed that JYQHD significantly suppressed GC cell viability and inhibited their proliferation. Collectively, it was demonstrated in this study that JYQHD effectively decreased the sphere size and number relative to the control group, indicating the role of JYQHD in inhibiting GC cell stemness. In recent studies, ferroptosis has been recognized to be the new cell death type related to metabolism, redox and iron-mediated lipid peroxidation [71]. During ferroptosis, Fe^2+^, ROS and MDA levels increase, while GSH content decreases, leading to cell death [72]. In the experimental results, JYQHD group exhibited higher ROS, MDA, Fe^2+^ contents whereas lower GSH level compared with the control group. Therefore, this study analyzed the impact of JYQHD on mitochondrial morphology. Not surprisingly, JYQHD significantly increased the mitochondrial membrane density and destroyed the mitochondrial ridge within MKN-45 and SGC-7901 cells under TEM, indicating that JYQHD promoted the ferroptosis of GC cells. The results suggested that JYQHD induced GC cell ferroptosis in vitro. To conclude, the JYQHD induced GC cell ferroptosis and inhibited GC cell stemness. This study provided a novel insight into the intervention of stemness and ferroptosis in GC by Chinese medicine.

The previous studies have shown that JYQHD can inhibit GC cell stemness and induce GC cell ferroptosis, while the link among them has not been clarified. Recent reports have explored the crossover and regulatory pathways controlling ferroptosis, which is the new method to target CSCs [73]. To elucidate the JYQHD-related mechanism in regulating cancer cell stemness in GC, this study targeted the ferroptosis pathway. To better determine the inhibitory effect of JYQHD-induced ferroptosis on stemness of cancer cells, Fer-1 was used to examine the presence/absence of the rescue effect. Consistent with previous results, JYQHD attenuated the cancer cell stemness and induced cell ferroptosis relative to the control group, while Fer-1 reversed the ferroptosis and the cancer cell stemness phenotypes induced by JYQHD. The *in-vitro* qRT-PCR and WB assays suggested the role of JYQHD in promoting ferroptosis by down-regulating GPX4 and SLC7A11 (markers for ferroptosis), while inhibiting the stemness by down-regulating Sox2 and Oct4 (markers for stemness). Therefore, Fer-1 abolished the changes in phenotypes and expression of markers for ferroptosis and stemness induced by JYQHD. Our studies have indicated that JYQHD inhibits GC stemness by down-regulating the expression of GPX4 and SLC7A11, activating the ferroptosis pathway, and thereby down-regulating the expression of Sox2 and Oct4. To sum up, this study illustrated that JYQHD made an anti-GC effect and attenuated cancer cell stemness by inducing the ferroptosis pathway in GC cells.

To obtain the anti-STAD targets in JYQHD, the TCMSP and PubChem databases were used to collect the active ingredients. The potential targets were predicted by Swiss Target Prediction website. The DisGeNET, GeneCards, and OMIM databases were used to obtain the relevant targets in GC. Finally, 355 overlapped genes between the predicted targets in JYQHD and the known therapeutic targets for STAD were obtained. Both GO and KEGG enrichment analysis results in 355 overlapped genes were associated with ferroptosis, which was predicted to be a potential mechanism of action of JYQHD against gastric cancer. In the 355 genes, Collagen family genes including COL1A1 and COL3A1 were found. To search for potential genes, we reviewed the literature. Shiping Liu et al. considered that the COL1A1-network might facilitate malignant metastasis and act as a prognostic marker in GC, and COL12A1 in this network might activate the EMT pathway [74]. Yihuan Chen et al. demonstrated that the collagen family, especially COL1A1, COL1A2, and COL12A1, might act as potential prognostic biomarkers and immune-associated therapeutic targets in gastric cancer [75]. Mengjun Li et al. revealed that the transcriptional and translational expression levels of the genes including COL1A1, COL5A2 and COL12A1 in Chinese GC tissues were higher than normal tissues [76]. Based on the literature research, we found that COL12A1 and COL1A1 were closely related. According to Network Pharmacology analysis, COL12A1 and COL1A1 were the target genes of gastric cancer. Meanwhile, COL1A1 was the target gene of JYQHD. In search of promising genes, we expanded the research scope to predict the value of COL1A1, COL2A1, COL3A1, COL4A1, COL5A1, COL6A1, COL7A1, COL8A1, COL9A1, COL10A1, COL11A1 and COL12A1. Through GEPIA database, we found that COL1A1, COL4A1 and COL12A1 were differentially expressed in patients with gastric cancer, significantly suggesting a poor prognosis. According to qRT-PCR, COL1A1, COL4A1 and COL12A1 mRNA expression elevated within GC cells compared with normal cells. Recently, COL12A1 has attracted increasing attention in tumor studies. Zengwei Tang et al. reported that COL12A1 was significantly upregulated in clinical intrahepatic cholangiocarcinoma (iCCA) tissue samples and was correlated with tumor stage. COL12A1-high expression was associated with the poor prognoses of iCCA patients. Experimental knockout of COL12A1 inhibited the proliferation, invasiveness and growth of iCCA cells [77]. Jiali Li et al. presented that METTL3 could promote cell proliferation, migration and invasion and upregulate the expression of COL12A1 in esophageal squamous cell carcinoma (ESCC) cell lines; besides, COL12A1 could restrain siMETTL3-mediated inhibition of proliferation, migration and invasion [78]. Yao Song et al. demonstrated the abnormally high expression of COL12A1 in pancreatic cancer and its clinical prognostic value in pancreatic cancer through the analysis of TCGA dataset. The knocking down of COL12A1 decreased the proliferation and migration of cancer-associated fibroblasts (CAFs) and down-regulated the expression of CAF activation markers [79]. Zhen Xiang et al. confirmed that IDO1 and COL12A1 synergistically promoted GC metastasis, and knockdown of COL12A1 inhibited GC cell migration [80]. Xiaoxia Jiang et al. suggested that the expression of COL12A1 was notably upregulated in GC. Subsequent clinicopathological analysis showed that elevated COL12A1 expression was positively correlated with tumor invasiveness, metastasis and advanced clinical stage. Meanwhile, the prognostic analysis indicated that COL12A1 overexpression contributed to poor overall survival in patients with GC [81]. Mihaela Chivu-Economescu et al. determined that COL12A1 was highly upregulated at the mRNA and protein levels and that the high expression of COL12A1 was correlated with poor overall survival in GC patients [82]. These results highlighted the importance of COL12A1 in a variety of malignancies, especially gastric cancer, and suggested its potential role as a candidate for clinical prognostic prediction and targeted therapy in patients with gastric cancer. However, there were few reports on the basic studies of COL12A1 in gastric cancer. Considering the value of this study, we selected COL12A1 as the target for subsequent research. COL1A1 and COL4A1 will be further investigated in future studies. Based on the bioinformatics analysis and literature search, we hypothesized that COL12A1 was one of the potential target genes of JYQHD, and COL12A1 could regulate the ferroptosis pathway in gastric cancer.

In this study, bioinformatics analysis was conducted to clarify the expression level and clinical significance of COL12A1 in gastric cancer, and in-vitro and in-vivo experiments were performed to further explore the role of COL12A1 in gastric cancer. COL12A1 mRNA expression increased in GC tissues compared with healthy tissues in TCGA-STAD, GSE13911 and GSE66229 datasets, as discovered from GEPIA database. As evidenced by analysis based on TCGA and Kaplan–Meier plotter databases, COL12A1 expression showed negative relationship with OS of GC cases. The result of the ROC curve demonstrated that the COL12A1 gene provided comparatively high diagnostic value for the patients of GC in the TCGA database. Moreover, according to HPA-based histochemical data, COL12A1 expression was up-regulated within GC tissues. Therefore, these findings indicated the vital effect of COL12A1 on promoting GC occurrence and progression. Elham Darang et al. elaborated that the importance of COL1A2, COL5A2, COL6A3 and COL12A1 in the development of the disease could be considered candidate genes in the prevention and early diagnosis of gastric cancer through bioinformatics and KEGG enrichment analysis [83]. However, the function of COL12A1 has not been completely developed. Aiming to explore the role of COL12A1 in GC, we conducted follow-up experiments. As a result, COL12A1 siRNA transfection suppressed the growth of the GC cell lines. Therefore, we further investigated the association of COL12A1 with ferroptosis and stemness in GC cells. In this study, the ferroptosis was promoted by decreasing COL12A1, and the stemness of GC cells was inhibited in vitro. Consistent with those within the in-vitro experiments, knockdown of COL12A1 in GC cells reduced the tumor mass growth and decreased the expression of GPX4 and SLC7A11 in xenograft models. These findings have provided evidence for the first time that COL12A1 promotes proliferation, enhances stemness, and inhibits ferroptosis in GC. These results explained the function of COL12A1 in gastric cancer, highlighting that COL12A1 might be an oncogene in GC.

To better explore the mechanism of JYQHD against GC and whether JYQHD achieved the anti-GC effect by inhibiting COL12A1, this study successfully over-expressed COL12A1 expression in MKN-45 and SGC-7901 cells. Consistent with the previous results, in-vitro results indicated that JYQHD could induce ferroptosis and inhibit stemness of GC cells. However, COL12A1 up-regulation obviously abolished the JYQHD-mediated activation of ferroptosis and suppression on stemness of GC cells compared with JYQHD group. Mechanistically, qRT-PCR and WB assays indicated that JYQHD inhibited COL12A1, GPX4, SLC7A11, Oct4 and Sox2 expressions, while overexpression of COL12A1 obviously enhanced the COL12A1, GPX4, SLC7A11, Oct4 and Sox2 expressions in relative to JYQHD group in vitro. In vivo, JYQHD suppressed xenograft tumor growth in GC, while COL12A1 overexpression promoted xenograft tumor growth and attenuated the anti-tumor effect of JYQHD. As revealed by IHC assay, JYQHD group displayed the reduced levels of COL12A1, GPX4 and SLC7A11, while an elevated levels of COL12A1, GPX4 and SLC7A11 were shown in JYQHD + oe-COL12A1 group. A previous study elaborated that Fuzheng Nizeng Decoction regulated ferroptosis and endoplasmic reticulum stress in the treatment of gastric precancerous lesions [84]. Our consequences presented that JYQHD activated the ferroptosis and inhibited the stemness in GC cell lines. COL12A1, ferroptosis markers and cancer stemness markers including GXP4, SLC7A11, Sox2 and Oct4 were obviously weakened by JYQHD. Moreover, overexpression of COL12A1 inhibited the ferroptosis phenotype, enhanced the stemness phenotype, and up-regulated the levels of GXP4, SLC7A11, Sox2 and Oct4. Hereby, we suggested that JYQHD promoted the ferroptosis, weakened the stemness, and controled the growth of subcutaneous grafts in xenograft models via COL12A1-mediated ferroptosis signaling pathway in GC cells. Moreover, our experiments provide novel evidence that JYQHD is a potential agent in gastric cancer therapy, and COL12A1 is a potentially valuable gene that regulates ferroptosis.

## Conclusions

To conclude, the obtained findings demonstrated that JYQHD hindered GC cell growth and stemness, induced ferroptosis in vitro, and controlled subcutaneous xenograft tumor growth in vivo. This study indicated a novel finding that COL12A1 inhibited the ferroptosis and enhanced the stemness in GC cells for the first time. Subsequently, these obtained findings illustrated the mechanism by which JYQHD down-regulated COL12A1, which then promoted the ferroptosis signaling pathway, and inhibited the stemness, ultimately playing an anti-GC role. Moreover, these findings provide experimental evidence for JYQHD as a TCM decoction that can act on human GC cells, thus promoting the development of novel anti-tumor drugs. A major limitation is the lack of research into the in-depth mechanisms and the active components. Future research should focus on further elucidating the underlying pharmacological mechanism.

### Supplementary Information

**Additional file 1. **All results of the overlapped genes between JYQHD and STAD target genes.

**Additional file 2. **All results of GO enrichment analysis of the overlapped genes.

**Additional file 3. **All results of KEGG enrichment analysis of the overlapped genes.

**Additional file 4: Table S1. **The death of nude mice.

## Data Availability

Network data can be found here: TCGA (https://portal.gdc.cancer.gov/), GEO (https://www.ncbi.nlm.nih.gov/gds), Kaplan–Meier plotter (http://kmplot.com/analysis/), GEPIA (http://gepia.cancer-pku.cn/), HPA (https://www.proteinatlas.org/), TCMSP (https://tcmsp-e.com/tcmsp.php), PubChem (https://pubchem.ncbi.nlm.nih.gov/), DisGeNET (https://www.disgenet.org/), GeneCards, OMIM (https://omim.org/), and Venn diagram (http://bioinformatics.psb.ugent.be/webtools/Venn/). Data utilized in the current work were contained in this study.

## Abbreviations

JYQHD: Jian Yun Qing Hua Decoction
GC: Gastric cancer
CCK8: Cell Counting Kit-8
MDA: Malondialdehyde
GSH: Glutathione
ROS: Reactive oxygen species
TEM: Transmission electron microscopy
qRT-PCR: Quantitative Real-Time Polymerase Chain Reaction
WB: Western-blot
IHC: Immunohistochemical
CSCs: Cancer stem cells
SLC3A2: Solute carrier family 3 member 2
SLC7A11: Subunit solute carrier family 7 member 11
GXP4: Glutathione peroxidase 4
COL12A1: Collagen type XII α1 chain
DEGs: Differentially expressed genes
TCM: Traditional Chinese Medicine
TLR4: Toll-Like Receptor 4
NF-κB: Nuclear factor-κB
LC-TOFMS: Liquid chromatography/time-of-flight mass spectrometry
FCS: Fetal calf serum
Fer-1: Ferrostatin-1
GEPIA: Gene Expression Profiling Interactive Analysis
GTEx: Genotype Tissue Expression
TCGA: The Cancer Genome Atlas
GEO: Gene Expression Omnibus
STAD: Stomach adenocarcinoma
ROC: Receiver operating characteristic
HPA: Human Protein Atla
KM: Kaplan–Meier
TCMSP: Traditional Chinese Medicine Systems Pharmacology
OMIM: Online Mendelian Inheritance in Man
GO: Gene Ontology
KEGG: Kyoto Encyclopedia of Genes and Genomes
CL: Confidence limits
OD: Absorbance
BPs: Biological processes
OS: Overall survival
AUC: Area under the curves
iCCA: Intrahepatic cholangiocarcinoma
ESCC: Esophageal squamous cell carcinoma
CAFs: Cancer-associated fibroblasts
