# Supplementary material for: Jian Yun Qing Hua Decoction inhibits malignant behaviors of gastric carcinoma cells via COL12A1 mediated ferroptosis signal pathway
Source: Chin Med. 2023 Sep 12;18:118. doi: 10.1186/s13020-023-00799-5 (PMC10496189; doi:10.1186/s13020-023-00799-5)
Supplement: Supplementary file 4 — Additional file 4: Table S1. The death of nude mice. [file 13020_2023_799_MOESM4_ESM.docx]

**Supplementary Information**

**Table S1**

The death of nude mice

| Diferent groups | Animals (number / group) | Drug administration (times / day) | Concentration (g / ml) | Volume (ml / 10 g) | Number of deaths (number / group) | Mortality rate (percent) |
| --- | --- | --- | --- | --- | --- | --- |
| Control group | 6 | 2 | - | 0.4 | 0 | 0 |
| Administration Group 1 | 6 | 2 | 1 | 0.4 | 0 | 0 |
| Administration Group 2 | 6 | 2 | 2 | 0.4 | 0 | 0 |
| Administration Group 3 | 6 | 2 | 3 | 0.4 | 0 | 0 |
| Administration Group 4 | 6 | 2 | 4 | 0.4 | 0 | 0 |
| Administration Group 5 | 6 | 2 | 5 | 0.4 | 0 | 0 |
